# Characteristics of Implementing Practice Development in Germany: A National Scoping Review

**DOI:** 10.1002/hsr2.70546

**Published:** 2025-03-18

**Authors:** Anne Fahsold, Theresa Siegler, Anna Louisa Hoffmann‐Hoffrichter, Sibylle Reick, Rebecca Palm

**Affiliations:** ^1^ Faculty of Health, Department of Nursing Science Witten/Herdecke University Witten Germany; ^2^ Klinikum rechts der Isar at the Technical University Munich München Germany; ^3^ Department of Health Services Research, School of Medicine and Health Sciences Carl von Ossietzky University of Oldenburg Oldenburg Germany

**Keywords:** complex intervention, nursing, practice development, review

## Abstract

**Background and Aims:**

Practice development is a clearly defined concept internationally. In Germany, however, the concept is understood differently. For this reason, it is unknown how the concept is implemented in patient care and whether it corresponds to the international concept. The aim was to create an overview of practice development projects in Germany and to examine whether the practice development projects included meet the internationally defined criteria for practice development.

**Methods:**

A scoping review was performed. For this purpose, a literature search was conducted in MEDLINE, CINAHL, CareLit, and GeroLit as well as by hand search in nonindexed German‐language journals. Articles were included in the evidence synthesis regardless of their methodological quality.

**Results:**

Eleven articles from 2012 to 2021 were included in the scoping review. The goals pursued by the projects focus on the implementation of evidence‐based nursing and advanced practice nursing, the integration of university‐trained nurses, the improvement of clinical care, and the competence development of nurses and leaders in the acute hospital setting. The review showed that few practice development projects incorporate the internationally recognized criteria of practice development and are based on the current international definition of practice development.

**Conclusion:**

It would appear beneficial and necessary to achieve a uniform understanding in Germany to undertake a process of concept clarification and to develop a definition of practice development for implementation.

## 1 Introduction

### 1.1 Rationale

Developments and efforts to improve patient care have been summarized in various terms. For example, *“nursing development,” “quality improvement,”* and *“practice development”* are commonly used terms when referring to nursing practice initiatives and projects. The latter term is currently gaining momentum in Germany, but the discourse in the scientific community also demonstrates that it is used inconsistently and that there are differences in its underlying meaning [1].

Parallels to today's situation in Germany can be seen by looking at the United Kingdom, where the term *“practice development”* was first used; Pearson established the definition in the context of the implementation of primary nursing in nursing development units [2]. Over the next two decades, the problem of inconsistent practice development approach use among colleagues emerged, even though a definition of practice development was first published in 1999 [3]; and updated in 2004 in the book “Practice Development in Nursing” [4]. Today, the internationally used definition is the following: *“Practice development is a continuous process of developing person‐centered cultures. It is enabled by facilitators who authentically engage with individuals and teams to blend personal qualities and creative imagination with practice skills and practice wisdom. The learning that occurs brings about transformations of individuals and team practices. This is sustained by embedding processes and outcomes in corporate strategy.”* (see reference [5]).

The initial concept clarification by Garbett and McCormack [6] led to the development of principles that underpinned the *“body of work”* of the core principles of practice development [7]. These methodological foundations and the definition of the concept were revised in subsequent years [4, 5, 8]. In addition, practice development methods and knowledge have advanced internationally. The International Practice Development Collaborative, which includes experts in practice development from Europe, North America, and Australia, was formed for this purpose [7].

For German‐speaking countries, the original definition and principles were translated and published in 2009 [9]. Publishing German language definitions, principles, and concepts provides a theoretical foundation for practice development activities in German‐speaking countries. The availability of these resources for practice development has been a significant step toward the establishment of a common theoretical foundation for individual activities in nursing practice. Thus far, it is unknown whether the current theoretical foundation of practice development is the basis for practice development activities, or whether other models, theories, and assumptions guide its implementation in Germany. Therefore, it is necessary to clarify which methodological understanding guides practice development in Germany and to establish a common framework for the goals of practice development. As a prelude to a conceptual definition [10, 11] of the term “*practice development”* in Germany, a literature review was conducted to gain an understanding of the ways in which practice development is being carried out and the activities associated with it.

### 1.2 Objectives

This review aims to provide an overview of practice development projects and initiatives in Germany and to examine the extent to which these projects meet the internationally defined criteria for practice development as a complex intervention [5, 12]. The following research questions serve to guide this objective:

*What are the defining characteristics of projects and initiatives of practice development in Germany?*

- *What are their intended outcomes?*
- *What are the principles that underpin the projects/initiatives?*
- *Which methods are employed?*

## 2 Methods

### 2.1 Design

To answer the research questions linked to this aim, we conducted a scoping review following the methodological recommendations of the Joanna Briggs Institute [13]. We used the *Population/Participants‐Concept‐Context* (PCC) mnemonic to structure the operationalization of our research questions and resulting search strategies [14]. Our scoping review is structured according to the PRISMA statement for scoping reviews (PRISMA‐ScR) [15]. There was no registration for this scoping review.

### 2.2 Information Sources and Search

A literature search in academic databases was conducted using MEDLINE via PubMed, and CINAHL via Ebsco. We also searched GeroLit and CareLit, two German databases indexing research, and gray literature. In addition, a hand search was conducted in different German nonacademic magazines and journals including *“intensive,” “Onkologische Pflege,” “PADUA,” “ProCare,” “Psychiatrie Pflege,”* and *“Schmerz und Schmerzmanagement,”* as these journals are not indexed in any database, but are of high relevance for nurses across German‐speaking countries [16]. The search strategies for individual databases were initially developed by two authors (Authors 2 and 3) and revised in collaboration with the entire team of authors. A sensitive search strategy, considering related keywords and subject headings, was applied to capture the diverse uses of the term *“practice development”* (Supporting Information 1). The search was conducted in June 2021 and updated in March 2022.

### 2.3 Eligibility Criteria

For this scoping review, the eligibility criteria listed in Table 1 were as follows: For *“population/participants,”* studies and project reports had to include a nursing perspective. Projects that were reported from a multidisciplinary perspective but did not include nurses were excluded. In terms of *“concept,”* authors had to explicitly refer to the reported project as a *“practice development project,”* as there are a variety of German terms that are used in a similar way, but do not have the same meaning from a conceptual perspective. We searched for practice development projects in all German health care settings but did not include other German‐speaking regions such as Switzerland, Austria, and Luxembourg, as developments and related conditions in the field of practice development may differ from those in Germany. The search was not limited in time, but was restricted to German and English. Publications that addressed practice development from a theoretical perspective (e.g., position papers) were excluded.

**Table 1:** Overview of eligibility criteria.

	Inclusion criteria	Exclusion criteria
Population/Participants	Nurses need to be part of the project team	Project studies without a nursing perspective
Concept	Projects and studies declared as “practice development projects”	Projects and studies that have been declared as some other kind of project in the context of health care (e.g., quality improvement projects)
Context	All health care settings in Germany	Health care settings in other countries than Germany
Sources of evidence	Literature including project and study reports, for example, Original research articles Magazine and nonacademic articles Project reports	Literature only focusing on a theoretical discourse of “practice development” or without empirical research reporting, for example, Discussion papers Handbooks Study protocols
Publication language	German, English	Other languages

### 2.4 Selection of Sources of Evidence

After removing all duplicates, articles were subjected to a multi‐step selection process using the online tool “*Rayyan*” [17]. First, the title‐abstract‐screening was independently performed by two authors (Authors 1 and 2). Articles for which there was no consensus regarding inclusion were reviewed by another author (Author 3). Full‐text screening was performed by one person (Author 1) because of limited personnel resources. If the application of the defined inclusion and exclusion criteria did not lead to a decision, Author 2 was consulted.

### 2.5 Data Charting Process and Synthesis of Results

Technology‐assisted data extraction using MAXQDA software [18] was performed by two researchers (Authors 1 and 2). The methodological recommendations for data extraction in qualitative evidence synthesized by Flemming and Noyes [19] were applied iteratively.

Data extraction was conducted using a deductive‐inductive approach, which defines practice development as a complex intervention in accordance with the Medical Research Council Framework [5]. To make these effects of practice development visible, they recommended describing the processes and outcomes using the criteria that every practice development project should have (Table 2) [5]. We used these criteria as a deductive basis for coding, and then developed inductive categories from the included reports. The team of authors discussed the results of coding procedures and data extraction.

**Table 2:** Criteria for practice development projects (data items).

Criteria questions to ascertain the use of practice development as a complex intervention and its process*		
Inclusion criteria: Identifying practice development projects/initiatives	a. Collaboration, inclusion, participation (CIP principles)	Which stakeholders were represented in your project study? How were stakeholders engaged in your project/study?
	b. Ethics	What ethical process were used with stakeholders/participants in your project/study?
	c. Shared ownership/vision	How did you develop shared ownership and a shared vision for your project/study?
	d. Facilitation	Who were the project facilitators? What approach to facilitation was used?
	e. Evaluation	How was the project/study evaluated?
Starting points to your practice development project/study	What was the starting point (purpose) for your project/study?	
	a. Values	Which values were focused on in your project/study?
	b. Workplace culture	How was workplace culture assessed implicitly or explicitly in your project/study?
Methodology/methods	What methods were used to facilitate critical reflection and learning?	
	What methods were used to promote and enable critical creativity?	
	What methods were used to help participants become practitioner‐researcher/inquiries into their own practice?	
	How was feedback and critical learning utilized?	

* Visualization of the authors according to Hardy and Manley in reference [3].

## 3 Results

**Figure 1:**
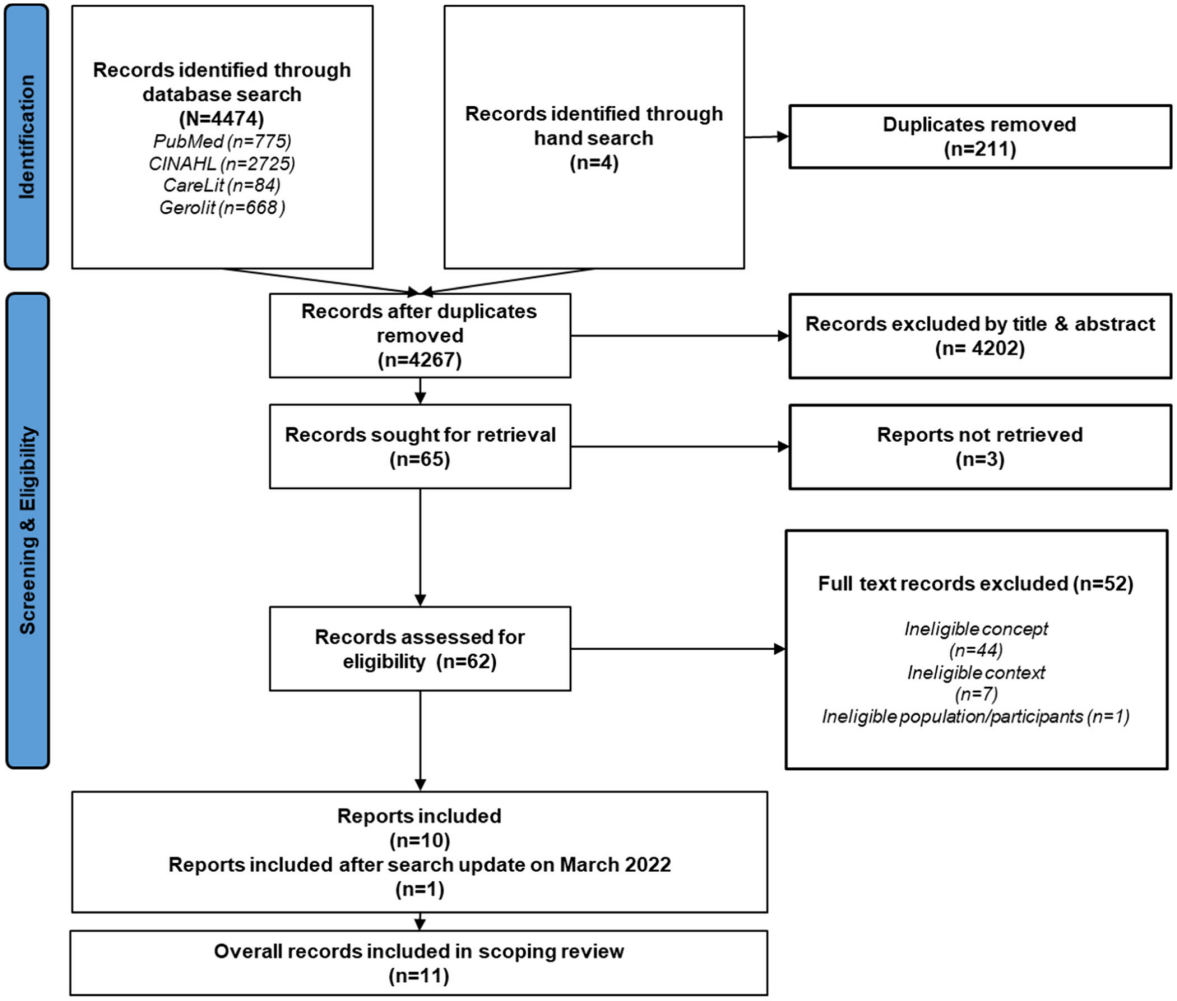
PRISMA flowchart according to Page et al. [20].

The search in the four databases and portals mentioned above, as well as the hand search, yielded 4478 hits. After removing duplicates, 4267 hits remained, of which we included 11 articles in the data synthesis (Figure 1). The full texts of the excluded articles and the reasons for their exclusion can be found in Supporting Information 2 (available only in German).

Table 3 presents an overview of the included articles, settings, aims, and project populations/participants. The articles were published between 2012 and 2021 in various formats and four reports were from peer‐reviewed journals [21, 22, 29, 31]. Among the projects included, the following can be distinguished: projects that pursue strategy development for the institution in the context of practice development [21, 22, 24, 27] and projects that focus on a specific topic, such as the implementation of advanced nursing practice in individual disciplines [23, 25, 26, 28, 29, 30, 31].

**Table 3:** Characteristics of included sources of evidence.

Authors/Year of publication	Setting	Focus of practice development	Aims of the project/study	Included profession/target groups
Bergjan and Bürger [21]	Maximum medical care clinic	Implementation of advanced nurse practitioners into “core teams”	Practice development by facilitating the implementation of evidence‐based interventions Integration of nurses at the bachelor level into practice	Nursing professionals with a bachelor degree
Eberhardt [22]	Specialized medical care clinic	Implementation of academic nursing roles	Expanding professional and role development toward a person‐centered approach to care Implementation of EBN** Systematic management and person‐centered implementation of the nursing process Task and role differentiated composition of care teams Development of leadership skills Development of a work and learning environment that promotes development Evidence of nursing outcomes Development of interprofessional collaboration and effective interface management	Nursing leaders Nursing professionals with a bachelor degree
Eberhardt [23]*	Specialized medical care clinic	Competence development of leaders for the promotion of practice development	Leadership skills building Develop awareness of roles and responsibilities Patient‐centered and evidence‐based nursing practice Facilitating culture and context change	Nursing leaders
Eberhardt and Wild [24]	Specialized medical care clinic	Evaluation of evidence‐based nursing implementation	Increase the effectiveness of patient‐centered care Increase EBN knowledge and competence Increase awareness of the concept of EBN Promote informed attitudes Specific planning of further dissemination/implementation activities	Nursing management Nursing education (faculty) Nursing practice (practice managers and hygiene officers)

Authors/Year of publication	Setting	Focus of practice development	Aims of the project/study	Target groups
Hock et al. [25]	Specialized medical care clinic	Possible applications of advanced practice nurses in neonatology	Improve skin care Develop an evidence‐based skin care guideline Strengthening the parent‐child bond Improve discharge management	Nursing professionals with a master's degree
Müller et al. [26]	Specialized medical care clinic	Implementation of nursing experts (master's level) in various disciplines	Function of APN nurse experts as a link between nursing practice and science Enhance team competency through tailored education	Not reported
Reick [27]	Specialized medical care clinic	Implementation of evidence‐based nursing	Evidence‐based nursing, including supporting nurses in a theory‐practice dialog Importance of effective leadership	Not reported
Schmidkonz and Eberhardt [28]	Specialized medical care clinic	Special postoperative exercise training for patients	Person‐centered care through patient engagement Increase independence after surgery	Registered nurses patients
Werner et al. [29]	Specialized medical care clinic	Review and evaluation regarding development process of nursing experts (master's level) including influencing factors	Require time and persistence for APN implementation Clarification of role for implementation essential	Not reported
Werneth et al. [30]	Specialized medical care clinic	Evidence‐based nutrition recommendations in pediatric oncology	Revision, implementation and evaluation of an information brochure “Nutrition” for oncological pediatrics Support for counseling/guidance of parents at initial diagnosis	Relatives/parents of oncology patients Pediatric oncology nurses
Wittmann et al. [31]	Specialized medical care clinic	Evaluation of nursing organization concept for patients with high case management needs	Systematic management and person‐centered implementation of the nursing process (a subset of the overall practice development strategy)	Not reported

* Consists of several consecutive and interrelated articles in a magazine; therefore, grouped together as one reference.

** Evidence‐based nursing.

### 3.1 Overview of Criteria Application for Practice Development Projects

Table 4 provides an overview of the key themes and knowledge gaps extracted from the included articles on adherence to internationally defined practice development criteria [5]. This process is outlined in detail in the following sections.

**Table 4:** Information about practice development criteria and patterning chart.

	Bergjan and Bürger [21]	Eberhardt [23]	Eberhardt [22]	Eberhardt and Wild [24]	Hock et al. [25]	Müller et al. [26]	Reick [27]	Schmidkonz and Eberhardt [28]	Werner et al. [29]	Werneth et al. [30]	Wittmann et al. [31]
Identification of project											
*CIP principles*	*x*	*x*	*x*	*x*	*x*	*x*	*x*	*x*	*x*	*x*	*x*
*Ethics*				*x*				*x*			
*Shared ownership/vision*		*x*	*xs*	*x*			*x*	*x*	*x*		
*Facilitation*		*x*	*x*	*x*			*x*	*x*	*x*		*x*
*Evaluation*	*x*	*x*	*x*	*x*	*x*	*x*	*x*	*x*	*x*	*x*	
Starting points											
*Values*		*x*	*x*	*x*			*x*	*x*			
*Workplace culture*			*x*	*x*				*x*	*x*		*x*
Methods											
*For reflection/learning*	*x*	*x*	x		*x*	*x*	*x*	*x*	*x*	*x*	*x*
*For critical creativity*		*x*	*x*	*x*	*x*		*x*	*x*			
*For practitioner‐learners*	*x*							*x*			
*For feedback*	*x*	*x*	*x*	*x*	*x*	*x*		*x*		*x*	*x*

### 3.2 Identification of Practice Development Projects

#### 3.2.1 Collaboration, Inclusion and Participation

All projects were identified as intra‐ and multi‐professional and involved people from management, nursing science/nursing development units, and teams from relevant departments. Nurses worked together with physicians, therapists, patients, and families. In addition, some teams collaborated with stakeholders from outside their institutions (e.g., nursing schools or pharmacies) [26]. The involvement of different stakeholders was achieved through (un)systematic methods of direct communication such as consultations, informal conversations, discussions, map queries, network meetings, and congresses. Indirect communication methods, such as written surveys, were also used.

### 3.3 Ethics

Some authors described actions to ensure compliance with ethical and privacy principles. For example, Schmidkonz and Eberhardt [28] informed the team about the purpose of an observation and the patients about the voluntariness, purpose, and anonymity of an interview, and obtained informed consent. Eberhardt and Wild [24] pointed out that interviews for formative evaluation were voluntary, but the questionnaires had to be assigned to a ward to derive measures. Participants' anonymity was ensured by not collecting any biographical data. Ethical approval was not obtained for any of the projects, partly because the data were available in an anonymized form and partly because there were primary internal purposes for conducting the projects (e.g., quality management). Wittmann et al. [31] consulted the Clinical Ethics Committee and the Data Protection Office.

### 3.4 Shared Ownership/Visions

The joint development of a vision and its communication were reported in six articles [22, 23, 24, 27, 28, 29]. For example, in the project described by Eberhardt [22], the development of a shared vision was participatory; all stakeholders should be aware of the strengths, weaknesses, opportunities, and threats. Schmidkonz and Eberhardt [28] called this shared value base the “Value House.”

### 3.5 Facilitation

In two articles, the projects were part of student theses [28, 30]. Therefore, we can assume that the authors were supervised. In addition, nurse scientists employed at the respective hospitals were involved. Eberhardt [23], Eberhardt [22], and Bergjan and Bürger [21] accompanied and steered the implementation of the practice development strategy in staff unit practice development and as the main responsible practice developers. The *“clinical nursing experts APN”* supported and reflected each other, and experienced advanced practice nurses helped to raise the profile in the respective department [26, 29]. It is clear that different individuals, from leaders to members of other professions partially involved in the project, have different approaches to facilitation: peer‐to‐peer consultation in the context of collaboration with advanced practice nurses [26, 29], fostering trust and relationships with empowering leaders [22], opportunities for personal reflection, and reflection on achieved goals at the organizational level. Further facilitation approaches can be identified in the support of role and task profiles, as well as in the inclusion of implementation processes (e.g., de‐escalation management).

### 3.6 Evaluation

To evaluate their practice development efforts, the authors used established models such as the PARIHS framework [32] or the Consolidated Framework For Implementation Research [26, 27, 29, 33]. Eberhardt and Wild [24] assessed evidence‐basing clinical nursing processes using a self‐developed assessment tool. The results were presented and reflected at the leadership level, and the extent to which the goals were achieved was assessed. The authors used formative evaluation so that the evaluation results could be used in the process to plan further implementation steps based on need. Formative and summative evaluations were conducted to evaluate the leadership program [23]. Formative evaluation was conducted using guided interviews that focused on the experience of program implementation. The summative evaluation focused on the degree of implementation according to the plan and assessment of leadership competencies. For their formative evaluation of nursing process management, Wittmann et al. [31] used qualitative interviews (with patients and team members) to identify influencing factors and perceived changes, and quantitative analysis of routine patient data to evaluate economic aspects.

### 3.7 Starting Points for Practice Development Projects

#### 3.7.1 Values

Leadership was addressed in seven articles. Leaders play a key role in communicating values. They act as pioneers by knowing their underlying values and representing them externally. In one clinic, the leadership program was the focus of practice development. A leadership program developed for hospitals was required for future practice developers. Eberhardt [23] and Schmidkonz and Eberhardt [28] referred to patient‐, staff‐, research‐, and outcome‐oriented leadership styles as one of their core values. They understood transformational leadership to be a condition for the development of emancipatory practices. Reick [27] identified effective leadership and patient‐centeredness as prerequisites for implementing evidence‐based nursing. Needs orientation and person‐centeredness were also reported [23, 24, 31].

### 3.8 Workplace Culture

Eberhardt [22] described testing the professional roles of academically trained nurses in practice development units (comparable to *nursing development units*). These units were committed to the continuous development of nursing practices and were supported by the management. According to the author, they provided the necessary environment for systematic testing, implementation, and evaluation of new nursing concepts. Other articles did not go into detail regarding whether and how workplace culture was evaluated.

## 4 Methods

### 4.1 Methods for Critical Reflection and Learning

Eberhardt and Wild [24] described how they developed a 1‐day educational offering that deepened as part of an internal leadership program. In addition, they created structures for continuous knowledge‐building by ensuring access to scientific databases and literature. To achieve their goals, they held reflective discussions with departments and team leaders about difficulties and support needs over the course of the project. They used a three‐step method: (1) raising awareness, (2) imparting knowledge, and (3) promoting commitment to and integrating evidence‐based nursing as a mandatory part of daily life. Actions to promote critical reflection included encouraging prospective observations using the crystal‐ball method in preparation for project implementation, identifying areas for improvement through patient interviews and nursing observations [28], and reviewing an education flyer based on evaluation [30]. Eberhardt [23] reported offering an 18‐month leadership program to further develop leadership skills. It included various learning activities, such as 360° feedback, mentoring, work shadowing, action learning, nursing observations, patient interviews, and team building. An initial summary showed that the interviews encouraged new ways of seeing and thinking. Workshops helped to break down traditional thinking patterns, challenge power structures, and develop one's own understanding of the profession. Other learning activities included journal clubs and continuing education [27].

### 4.2 Methods to Promote Critical Creativity

We assigned the value assessment of Schmidkonz and Eberhardt [28] to the methods for fostering critical creativity. For this purpose, postcards were first formulated, read aloud, and analyzed, after which the values were discussed and concretized. From these results, goals were derived and a “Value House” was developed and presented.

### 4.3 Methods to Help Participants to Become Practitioner‐Researcher

The following methods were used to examine their own practices: strength‐weakness and opportunity‐risk analyses [22, 28] and situational analyses [21, 25, 26].

### 4.4 Methods for Feedback and Critical Learning

In the included projects, feedback was obtained, for example, through the presentation of complex patient cases [27] or the observation of nursing actions [23] to reflect on nursing activities. Wittmann et al. [31] also reported on case reflections as part of the competence training of case managers.

## 5 Discussion

This scoping review presents a sample of the practice development projects conducted in Germany. Based on a systematic search of four databases and an extensive hand search of nursing journals, 11 articles on practice development projects were identified. These were reviewed to determine whether they met internationally accepted criteria for practice development as complex interventions. All the projects were conducted in an acute care setting. The goals of the projects focused on the implementation of evidence‐based nursing and advanced nursing practice, integration of academically trained nurses, improvement of clinical care, and the development of nursing and leadership competencies.

Looking at projects in terms of the criteria for practice development as a complex intervention [5], some criteria were included in the vast majority of projects, whereas others were vague or not reported at all. For example, of the criteria identified by Hardy and Manley [5] for identifying practice development projects, the criteria “collaboration‐inclusion‐participation,” “shared ownership/vision,” and “facilitation” were included in the vast majority of projects. In contrast, the criteria “ethics” and “evaluation” were reported in only a few projects. The reasons these criteria were not considered need to be elucidated.

Ethics may play a subordinate role because the practice development projects were not research projects but projects that can be assigned to the institution's internal quality management. Not all projects collected data or involved patients directly. Thus, the question of ethical responsibility does not necessarily arise.

We attribute the result that the aspect of “evaluation” is only reported in detail in a few articles to the fact that in the respective (nonscientific) publication media, the evaluation method of practice development is given less attention than the lived experiences. However, we consider the nonreporting of evaluation methods and results problematic because it means that the effectiveness of practice development cannot be scientifically proven and is only reported anecdotally. Several tools such as the Workplace Culture Critical Analysis Tool (WCCAT) [34], that can be used to describe practice development in a visible and objective way. These methods should be used to make the practice development process transparent and understandable.

The description of the starting point for practice development projects was vague in most publications. Among core values, leadership received the most attention. This is also in line with current trends in international practice development [35]; the area of *“person‐centeredness, patient‐centeredness, and needs‐centeredness”* was considered in only a few projects, again, a major discrepancy with international practice development. Workplace culture was described in almost no project reports. In the international approach, the latter is an important aspect and is the focus of many practice development projects. For this reason, the discrepancy between the international concept and implementation of practice development in Germany is considered a key finding that requires further attention.

The methodology used in practice development projects has been described in detail by a few authors. However, we consider these descriptions particularly valuable for practice development, as they can be replicated by others and allow for a focused exchange of practice development methods.

The fact that the elements were not reported does not mean that they were irrelevant; however, in our view, relevance must be critiqued in reporting. For example, the consideration of workplace culture is essential for the sustainability of practice development. Different subcultures in organizations need to be included [36] to be successful in the nonlinear process of practice development in the long run. Fundamentally, nonreporting of knowledge generated in projects exacerbates the problem of implicit knowledge, which is generally prevalent in nursing [37].

The results allow us to draw conclusions about the current focus of practice development in Germany, which can be exemplified by the implementation of specialized and extended clinical nurses. For example, three of the four hospitals included in the study report how role profiles for nurses have been developed and implemented so that these individuals could subsequently act as facilitators. These specialized nurses assume professional leadership roles in their subsequent work. Thus, practice development has also been shown to be helpful in developing an organizational culture for transformational leadership [38].

## 6 Limitations

The results presented here must be critically evaluated against the following limitations: The articles included do not represent practice development activities in Germany in their entirety, but can only be evaluated as a sample. This is because most practice development projects in Germany are not published, and therefore, cannot be examined in the context of a literature review. Another limitation of this study is that details about the practice development criteria were not published in depth; therefore, sometimes during data extraction, the anticipation of their content was limited. Only articles that explicitly identified their project as a practice development project were included in the scoping review. Projects with similar names, such as *“practice or care project,”* were not included. We chose this limitation to ensure that all projects included had the concept of practice development as their basis, at least theoretically. In addition, certain aspects of the projects, such as the delineation between goals and outcomes already achieved, could not be presented in depth because of heterogeneous reporting. No projects were found outside the acute hospital setting; therefore, the results can only be used to draw conclusions in this setting.

## 7 Conclusions

For the overarching concept analysis, this scoping review provides an initial overview of the goals, characteristics, principles, and outcomes of practice development projects and initiatives in Germany. It also explores the theoretical underpinnings of these projects. These findings can be used as a basis for further empiricism and for identifying commonalities or differences between explicit and implicit practice development plans. Owing to the lack of consistency in reporting practice projects, the definition of practice development projects in Germany remains unclear. We suggest the development of reporting guidelines for practice development projects to improve and ease the reporting of projects conducted in nursing practice.

Furthermore, the implications of the individual characteristics of the included projects must be considered. For example, there is a need to further explore and capture nurses' leadership competencies using professional leadership and shared governance approaches. The dissemination of practice knowledge about facilitators and barriers to practice development should be pursued. Standardized reporting based on the characteristics, principles, and methods of practice development could help clarify the concept and classify the status of implementation actions carried out in practice development projects in Germany. Aspects such as workplace culture and shared values disseminate knowledge that can assist practice developers in the process management of their initiatives. To reduce the financial and time barriers linked to these efforts, dissemination channels such as websites, blogs, and social media could be beneficial.

## Author Contributions

Anne Fahsold: conceptualization, data curation, formal analysis, investigation, project administration, resources, software, visualization, writing – original draft, writing – review and editing. Theresa Siegler: conceptualization, formal analysis, investigation, methodology, resources, software, writing – original draft, writing – review and editing. Anna Louisa Hoffmann‐hoffrichter: conceptualization, methodology, supervision, validation, writing – review and editing. Sibylle Reick: conceptualization, resources, supervision, validation, writing – review and editing. Rebecca Palm: conceptualization, methodology, project administration, supervision, validation, writing – review and editing.

## Conflicts of Interest

The authors declare no conflicts of interest.

## Transparency Statement

The lead author Anne FAHSOLD affirms that this manuscript is an honest, accurate, and transparent account of the study being reported; that no important aspects of the study have been omitted; and that any discrepancies from the study as planned (and, if relevant, registered) have been explained.

## Supporting information

Supporting information.

Supporting information.

Supporting information.

## Data Availability

The data that support the findings of this study are available from the corresponding author upon reasonable request.
