# Supplementary material for: Characteristics of Implementing Practice Development in Germany: A National Scoping Review
Source: Health Sci Rep. 2025 Mar 18;8(3):e70546. doi: 10.1002/hsr2.70546 (PMC11915117; doi:10.1002/hsr2.70546)
Supplement: Supplementary file 1 — Supporting information. [file HSR2-8-e70546-s002.docx]

**SUPPLEMENT 1. Search strategies**

| **PubMed** | | | **Initial search conducted on: 11.06.2021** |
| --- | --- | --- | --- |
|  |  |  | **Search updated on: 01.03.2022** |
| ((((("health care quality, access, and evaluation/nursing"[MeSH Terms] OR "health care quality, access, and evaluation/organization and administration"[MeSH Terms] OR "health care quality, access, and evaluation/standards"[MeSH Terms] OR "health care quality, access, and evaluation/trends"[MeSH Terms] OR ("program development/methods"[MeSH Terms] OR "program development/organization and administration"[MeSH Terms] OR "program development/standards"[MeSH Terms]) OR ("staff development/methods"[MeSH Terms] OR "staff development/organization and administration"[MeSH Terms] OR "staff development/standards"[MeSH Terms] OR "staff development/trends"[MeSH Terms]) OR ("research/education"[MeSH Terms] OR "research/organization and administration"[MeSH Terms]) OR ("professional competence/organization and administration"[MeSH Terms] OR "professional competence/standards"[MeSH Terms]) OR ("clinical competence/education"[MeSH Terms] OR "clinical competence/organization and administration"[MeSH Terms] OR "clinical competence/standards"[MeSH Terms] OR "clinical competence/trends"[MeSH Terms]) OR "Social Skills"[MeSH Terms] OR ("cultural competency/education"[MeSH Terms] OR "cultural competency/organization and administration"[MeSH Terms]) OR "social change/organization and administration"[MeSH Terms] OR "Organizational Innovation"[MeSH Terms] OR "Organizational Culture"[MeSH Terms] OR ("learning/education"[MeSH Terms] OR "learning/organization and administration"[MeSH Terms] OR "learning/standards"[MeSH Terms] OR "learning/trends"[MeSH Terms]) OR ("group processes/education"[MeSH Terms] OR "group processes/organization and administration"[MeSH Terms] OR "group processes/trends"[MeSH Terms]) OR "Career Mobility"[MeSH Terms] OR ("clinical nursing research/education"[MeSH Terms] OR "clinical nursing research/organization and administration"[MeSH Terms] OR "clinical nursing research/standards"[MeSH Terms] OR "clinical nursing research/trends"[MeSH Terms]) OR ("nursing evaluation research/education"[MeSH Terms] OR "nursing evaluation research/methods"[MeSH Terms] OR "nursing evaluation research/organization and administration"[MeSH Terms] OR "nursing evaluation research/standards"[MeSH Terms] OR "nursing evaluation research/trends"[MeSH Terms]) OR ("education, nursing/education"[MeSH Terms] OR "education, nursing/methods"[MeSH Terms] OR "education, nursing/organization and administration"[MeSH Terms] OR "education, nursing/standards"[MeSH Terms] OR "education, nursing/trends"[MeSH Terms]) OR ("learning/analysis"[MeSH Terms] OR "learning/education"[MeSH Terms] OR "learning/methods"[MeSH Terms] OR "learning/organization and administration"[MeSH Terms] OR "learning/standards"[MeSH Terms] OR "learning/trends"[MeSH Terms]) OR ("quality improvement/organization and administration"[MeSH Terms] OR "quality improvement/standards"[MeSH Terms] OR "quality improvement/trends"[MeSH Terms]) OR "Social Facilitation"[MeSH Terms] OR ("parish nursing/education"[MeSH Terms] OR "parish nursing/methods"[MeSH Terms] OR "parish nursing/organization and administration"[MeSH Terms] OR "parish nursing/standards"[MeSH Terms] OR "parish nursing/trends"[MeSH Terms]) OR "Social Support"[MeSH Terms] OR ("mentors/education"[MeSH Terms] OR "mentors/organization and administration"[MeSH Terms]) OR ("counseling/education"[MeSH Terms] OR "counseling/methods"[MeSH Terms] OR "counseling/organization and administration"[MeSH Terms] OR "counseling/standards"[MeSH Terms] OR "counseling/trends"[MeSH Terms]) OR ("problem based learning/methods"[MeSH Terms] OR "problem based learning/organization and administration"[MeSH Terms] OR "problem based learning/standards"[MeSH Terms] OR "problem based learning/trends"[MeSH Terms]) OR "Nurse's Role"[MeSH Terms] OR ("advanced practice nursing/methods"[MeSH Terms] OR "advanced practice nursing/organization and administration"[MeSH Terms] OR "advanced practice nursing/standards"[MeSH Terms] OR "advanced practice nursing/trends"[MeSH Terms]) OR "Problem Solving"[MeSH Terms] OR "Cooperative Behavior"[MeSH Terms] OR "Intersectoral Collaboration"[MeSH Terms] OR "Interprofessional Relations"[MeSH Terms] OR "Social Participation"[MeSH Terms] OR "Stakeholder Participation"[MeSH Terms] OR ("primary nursing/education"[MeSH Terms] OR "primary nursing/methods"[MeSH Terms] OR "primary nursing/organization and administration"[MeSH Terms] OR "primary nursing/standards"[MeSH Terms] OR "primary nursing/trends"[MeSH Terms]) OR ("patient centered care/methods"[MeSH Terms] OR "patient centered care/organization and administration"[MeSH Terms] OR "patient centered care/standards"[MeSH Terms] OR "patient centered care/trends"[MeSH Terms]) OR ("patient education as topic/methods"[MeSH Terms] OR "patient education as topic/organization and administration"[MeSH Terms] OR "patient education as topic/standards"[MeSH Terms] OR "patient education as topic/trends"[MeSH Terms]) OR ("holistic nursing/methods"[MeSH Terms] OR "holistic nursing/organization and administration"[MeSH Terms] OR "holistic nursing/standards"[MeSH Terms] OR "holistic nursing/trends"[MeSH Terms]) OR "Empathy"[MeSH Terms] OR ("patient participation/methods"[MeSH Terms] OR "patient participation/organization and administration"[MeSH Terms] OR "patient participation/trends"[MeSH Terms]) OR ("culture/nursing"[MeSH Terms] OR "culture/trends"[MeSH Terms]) OR ("evidence based nursing/education"[MeSH Terms] OR "evidence based nursing/methods"[MeSH Terms] OR "evidence based nursing/organization and administration"[MeSH Terms] OR "evidence based nursing/standards"[MeSH Terms] OR "evidence based nursing/trends"[MeSH Terms]) OR "evidence based practice"[MeSH Terms] OR "decision making, shared"[MeSH Terms] OR "environment/nursing"[MeSH Terms] OR "environment/organization and administration"[MeSH Terms] OR "risk management/methods"[MeSH Terms] OR "risk management/organization and administration"[MeSH Terms] OR "risk management/trends"[MeSH Terms] OR "Patient Preference"[MeSH Terms] OR "Social Values"[MeSH Terms] OR "philosophy, nursing/education"[MeSH Terms] OR "philosophy, nursing/methods"[MeSH Terms] OR "philosophy, nursing/organization and administration"[MeSH Terms] OR "philosophy, nursing/standards"[MeSH Terms] OR "philosophy, nursing/trends"[MeSH Terms]) AND "german*"[All Fields] AND ("nursing"[MeSH Terms] OR "breast feeding"[MeSH Terms])) OR ("strategy"[Title/Abstract] OR "shared value*"[Title/Abstract] OR "philosophy"[Title/Abstract] OR "vision"[Title/Abstract] OR "social value*"[Title/Abstract] OR "physical environment"[Title/Abstract] OR "risk management"[Title/Abstract] OR "workplace environment"[Title/Abstract] OR "transformational leadership"[Title/Abstract] OR "shared governance"[Title/Abstract] OR "shared leadership"[Title/Abstract] OR "power sharing"[Title/Abstract] OR "organizational support*"[Title/Abstract] OR ("qualificat*"[Title/Abstract] AND ("nursing"[MeSH Terms] OR "nursing"[All Fields] OR "nursings"[All Fields] OR "nursing"[MeSH Subheading] OR "breast feeding"[MeSH Terms] OR ("breast"[All Fields] AND "feeding"[All Fields]) OR "breast feeding"[All Fields] OR "nursing s"[All Fields]) AND "german*"[All Fields]) OR "qualificat*"[Title/Abstract] OR "skill mix"[Title/Abstract] OR "practice change"[Title/Abstract] OR "organizational development"[Title/Abstract] OR "shared decision making*"[Title/Abstract] OR "collaborative decision*"[Title/Abstract] OR "development cultur*"[Title/Abstract] OR "transform cultur*"[Title/Abstract] OR "cultural competency"[Title/Abstract] OR "nursing culture"[Title/Abstract] OR "organizational cultur*"[Title/Abstract] OR "team cultur*"[Title/Abstract] OR "work cultur*"[Title/Abstract] OR "workplace cultur*"[Title/Abstract] OR "nursing research"[Title/Abstract] OR "ebp"[Title/Abstract] OR "evidence based practice"[Title/Abstract] OR "ebn"[Title/Abstract] OR "evidence-based nursing"[Title/Abstract] OR "patient participation"[Title/Abstract] OR "patient involv*"[Title/Abstract] OR "patient collaboration"[Title/Abstract] OR "empath*"[Title/Abstract] OR "holistic care"[Title/Abstract] OR "family education"[Title/Abstract] OR "patient education"[Title/Abstract] OR "person centred care"[Title/Abstract] OR "primary nurs*"[Title/Abstract] OR "counselors"[Title/Abstract] OR "participation"[Title/Abstract] OR "relationship"[Title/Abstract] OR "empower*"[Title/Abstract] OR "reflection*"[Title/Abstract] OR "cooperat*"[Title/Abstract] OR "collaborat*"[Title/Abstract] OR "teamwork"[Title/Abstract] OR "problem-based learning"[Title/Abstract] OR "problem solv*"[Title/Abstract] OR "advanced nurse practitioner"[Title/Abstract] OR "advanced practice nurs*"[Title/Abstract] OR "APN"[Title/Abstract] OR "ANP"[Title/Abstract] OR "role development"[Title/Abstract] OR "change agent*"[Title/Abstract] OR "case review*"[Title/Abstract] OR "guidance"[Title/Abstract] OR "advise*"[Title/Abstract] OR "consult*"[Title/Abstract] OR "support*"[Title/Abstract] OR "accompan*"[Title/Abstract] OR "companionship"[Title/Abstract] OR "facilitate"[Title/Abstract] OR "facilitation"[Title/Abstract] OR "quality development"[Title/Abstract] OR "quality improvement*"[Title/Abstract] OR "practice learning"[Title/Abstract] OR "practice education"[Title/Abstract] OR "parihs*"[Title/Abstract] OR "practice evaluation"[Title/Abstract] OR "practice theory"[Title/Abstract] OR "innovation*"[Title/Abstract] OR "alteration*"[Title/Abstract] OR "organizational change"[Title/Abstract] OR "transform*"[Title/Abstract] OR "competence*"[Title/Abstract] OR "staff development"[Title/Abstract] OR "profession development"[Title/Abstract] OR "practice development"[Title/Abstract]) OR "program development"[Title/Abstract] OR "clinical competence"[Title/Abstract] OR "social change"[Title/Abstract] OR "professional competence"[Title/Abstract] OR "Social Support"[Title/Abstract] OR "mentor*"[Title/Abstract] OR "nursing environment"[Title/Abstract]) AND ("organizational commitment"[Title/Abstract] OR "effective patient care"[Title/Abstract] OR "nursing outcome"[Title/Abstract] OR "organizational outcome"[Title/Abstract] OR "Organizational Culture"[Title/Abstract] OR "benchmark"[Title/Abstract] OR "Professional Autonomy"[Title/Abstract] OR "Knowledge"[Title/Abstract] OR "quality improvement"[Title/Abstract] OR "workflow*"[Title/Abstract] OR "flourish*"[Title/Abstract] OR "creativ*"[Title/Abstract] OR "empower*"[Title/Abstract] OR "emancipat*"[Title/Abstract] OR "Empathy"[Title/Abstract] OR "connected*"[Title/Abstract] OR "commitment"[Title/Abstract] OR "Work Engagement"[Title/Abstract] OR "Job Satisfaction"[Title/Abstract] OR "personal satisfaction"[Title/Abstract] OR "Patient Outcome Assessment"[Title/Abstract] OR "patient comfort"[Title/Abstract] OR "patient care"[Title/Abstract] OR "patient quality"[Title/Abstract] OR "Patient Satisfaction"[Title/Abstract] OR "patient outcome*"[Title/Abstract] OR "patient cultur*"[Title/Abstract] OR "patient safety"[Title/Abstract] OR "wellbeing"[Title/Abstract] OR "health care quality"[Title/Abstract] OR "quality improvement*"[Title/Abstract] OR "quality indicator*"[Title/Abstract] OR "quality indicators"[Title/Abstract] OR "Quality of Life"[Title/Abstract] OR ("Patient Reported Outcome Measures"[MeSH Terms] OR "quality improvement/organization and administration"[MeSH Terms] OR "quality improvement/standards"[MeSH Terms] OR "quality improvement/trends"[MeSH Terms] OR "quality indicators, health care/methods"[MeSH Terms] OR "quality indicators, health care/organization and administration"[MeSH Terms] OR "quality indicators, health care/standards"[MeSH Terms] OR "quality indicators, health care/trends"[MeSH Terms] OR "Quality of Life"[MeSH Terms] OR "Professional Autonomy"[MeSH Terms] OR "patient safety/organization and administration"[MeSH Terms] OR "patient safety/standards"[MeSH Terms] OR "patient safety/statistics and numerical data"[MeSH Terms] OR "Patient Outcome Assessment"[MeSH Terms] OR "Patient Satisfaction"[MeSH Terms] OR "Job Satisfaction"[MeSH Terms] OR "Work Engagement"[MeSH Terms] OR "Social Identification"[MeSH Terms] OR "Empathy"[MeSH Terms] OR "Creativity"[MeSH Terms] OR "Workflow"[MeSH Terms] OR "Knowledge"[MeSH Terms] OR "benchmarking/methods"[MeSH Terms] OR "benchmarking/organization and administration"[MeSH Terms] OR "benchmarking/standards"[MeSH Terms] OR "benchmarking/statistics and numerical data"[MeSH Terms] OR "benchmarking/trends"[MeSH Terms]) OR "Knowledge"[MeSH Terms] OR "Knowledge"[MeSH Terms]) AND "german*"[Affiliation] AND ("nursing"[MeSH Terms] OR "breast feeding"[MeSH Terms] OR ("nursing"[MeSH Terms] OR "nursing"[All Fields] OR "nursings"[All Fields] OR "nursing"[MeSH Subheading] OR "breast feeding"[MeSH Terms] OR ("breast"[All Fields] AND "feeding"[All Fields]) OR "breast feeding"[All Fields] OR "nursing s"[All Fields]))) NOT "midwife*"[All Fields]) AND (english[Filter] OR german[Filter])  (german*[Affiliation])) | | | |
| **CINAHL** | **Initial search conducted on: 11.06.2021** | | |
|  | **Search updated on: 01.03.2022** | | |
| ( (nurs* or care*) AND ( (TI “practice development” OR AB “practice development”) OR (TI “profession development” OR AB “profession development”) OR (TI “staff development*” OR AB “staff development*”) OR (TI transform* OR AB transform*) OR (TI “practice change*” OR AB “practice change*”) OR (TI “organizational change” OR AB “organizational change”) OR (TI alteration* OR AB alteration*) OR (TI innovation* OR AB innovation*) OR (TI “practice theory*” OR AB “practice theory*”) OR (TI “practice evaluation” OR AB “practice evaluation”) OR (TI PARIHS* OR AB PARIHS*) OR (TI “practice education” OR AB “practice education”) OR (TI “practice learning” OR AB “practice learning”) OR (TI “quality improvement*” OR AB “quality improvement*”) OR (TI “quality development” OR AB “quality development”) OR (TI “behavioral objectives in nursing” OR AB “behavioral objectives in nursing”) OR (TI “behavioral objectives*” OR AB “behavioral objectives*”) OR (TI “clinical competenc*” OR AB “clinical competenc*”) OR (TI "nursing skills*" OR AB "nursing skills*") OR (TI "cultural competence" OR AB "cultural competence") OR (TI "professional competence" OR AB "professional competence") OR (TI "learning environment" OR "learning environment") OR (TI "learning organization" OR AB "learning organization") OR (TI "learning method*" OR AB "learning method*") OR (TI "experiential learning*" OR AB "experiential learning*") OR (TI "self directed learning" OR AB "self directed learning") OR (TI "program development" OR AB "program development") OR (TI "program evaluation" OR AB "program evaluation") OR (TI "program implementation" OR AB "program implementation") OR (TI "program planning" OR AB "program planning") OR (TI "employee orientation" OR AB "employee orientation") OR (TI "professional recognition" OR AB "professional recognition") OR (TI "lifelong learning nursing" OR AB "lifelong learning nursing") OR (TI "promotion and tenure" OR AB "promotion and tenure”) OR (TI "problem identification" OR AB "problem identification") OR (TI "problem solving" OR AB "problem solving") OR (TI "goals and objectives" OR AB "goals and objectives") OR (TI "diffusion of innovation" OR AB "diffusion of innovation") OR (TI "theory practice relationship" OR AB "theory practice relationship") OR (TI "professional practice*" OR AB "professional practice*") OR (TI "nursing practice*" OR AB "nursing practice*") OR (MH "Behavioral Objectives/ED/OG/EV/PC/TD/ST") OR (MH "Clinical Competence") OR (MH "Nursing Skills") OR (MH "Cultural Competence") OR (MH "Professional Competence/ED/EV/OG/ST/TD/UT")OR (MH "Learning Methods") OR (MH "Experiential Learning") OR (MH "Self Directed Learning/ED/EV/MT/OG/ST/TD/UT") OR (MH "Program Development") OR (MH "Program Implementation") OR (MH "Program Planning/ED/EV/MT/OG/ST/TD/UT") OR (MH "Staff Development/ED/EV/MT/OG/ST/TD/UT") OR (MH "Employee Orientation/ED/EV/MT/OG/ST/TD/UT") OR (MH "Professional Development+/ED/EV/MT/ST/TD/UT") OR (MH "Professional Recognition") OR (MH "Professionalism") OR (MH "Promotion and Tenure/EV/MT/OG/ST/TD/UT") OR (MH "Lifelong Learning/ED/EV/MT") OR (MH "Problem Identification/EV/MT") OR (MH "Problem Solving+/ED/EV") OR (MH "Program Evaluation/ED/EV/MT/OG/ST/TD/UT") OR (MH "Goals and Objectives+") OR (MH "Diffusion of Innovation+/ED/EV/MA/MT") OR (MH "Theory-Practice Relationship+/ED/EV/MT/OG/ST/TD/UT") OR (MH "Professional Practice+/ED/EV/MA/MT/ST/TD/UT") OR (MH "Learning Environment, Clinical") OR (MH "Learning Laboratories") OR (MH "Learning Environment/ED/EV/OG/ST/TD/UT") OR (MH "Nursing Practice, Theory-Based/ED/EV/MA/MT/OG/ST/TD/UT") OR (TI facilition OR AB facilition) OR (TI facilitate OR AB facilitate) OR (TI facilitators OR AB facilitators) OR (TI companionship OR companionship) OR (TI accompan* OR AB accompan*) OR (TI support* OR support*) OR (TI consult* OR AB consult*) OR (TI membership* OR AB membership*) OR (TI mentor* OR AB mentor*) OR (TI advis* OR AB advis*) OR (TI guidance* OR AB guidance*) OR (TI “case review*” OR AB “case review*”) OR (TI “change agent*” OR AB “change agent*”) OR (TI "clinical supervision" OR AB "clinical supervision") OR (TI “role development” OR AB “role development”) OR (TI "advanced practice nurse" OR AB "advanced practice nurse") OR (TI "advanced practice nursing" OR AB "advanced practice nursing") OR (TI "nurse practitioner*" OR AB "nurse practitioner*") OR (TI “role model*” OR AB “role model*”) OR (TI “problem solv*” OR “problem solv*”) OR (TI “problem based learning” OR AB “problem based learning”) OR (TI teamwork OR AB teamwork) OR (TI collaborat* OR AB collaborat*) OR (TI cooperat* OR AB cooperat*) OR (TI reflection* OR AB reflection*) OR (TI empower* OR AB empower*) OR (TI relationship* OR AB relationship*) OR (TI participation OR AB participation) OR (TI councelling* OR AB councelling*) OR (TI fieldwork* OR AB fieldwork*) OR (TI "education in nursing" OR AB "education in nursing") OR (TI "internship and residency" OR AB "internship and residency") OR (TI "education model" OR AB "education model") OR (TI teaching* OR AB teaching*) OR (TI "journal clubs in nursing" OR AB "journal clubs in nursing") OR (TI "knowledge management" OR AB "knowledge management") OR (TI "conflict management" OR AB "conflict management") OR (TI "interprofessional relations" OR AB "interprofessional relations") OR (MH "Education, Clinical") OR (MH "Clinical Supervision") OR (MH "Clinical Supervision, Mental Health") OR (MH "Fieldwork/ST/OG/EV/ED/UT") OR (MH "Clinical Supervision") OR (MH "Clinical Supervision, Mental Health") OR (MH "Fieldwork/ST/OG/EV/ED/UT") OR (MH "Education, Nursing, Theory-Based") OR (MH "Entry Into Practice") OR (MH "Internship and Residency/ED/EV/OG/UT/TD/ST") OR (MH "Communication Skills Training") OR (MH "Teaching") OR (MH "Models, Educational") OR (MH "Social Skills Training/ED/EV/OG/ST/TD/UT") OR (MH "Teaching Materials, Clinical/ED/EV/OG/ST/TD/UT") OR (MH "Journal Clubs/ED/OG/TD/UT") OR (MH "Teaching Methods, Clinical+/ED/EV/MA/TD") OR (MH "Power+/ED/EV/TD") OR (MH "Mentorship/ED/EV/MA/MT/TD/UT") OR (MH "Cross Training/ED/EV/MT/OG/ST/TD/UT") OR (MH "Knowledge Management/EV/MA/MT/OG/ST/TD/UT") OR (MH "Conflict Management/ED/EV/MT/UT") OR (MH "Collaboration/ED/EV/MT/ST/TD/UT") OR (MH "Teamwork/ED/MT/ST/TD/UT") OR (MH "Interprofessional Relations/ED/EV/TD") OR (MH "Intraprofessional Relations/ED/EV/TD") OR (MH "Social Integration/ED/EV") OR (MH "Membership/ED/EV/MT/MA/ST/SN/TD/UT") OR (MH "Support Groups/ED/EV/MA/MT/UT") OR (MH "Role Models/ED/EV/ED /EV /OG /TD") OR (MH "Advanced Nursing Practice+") OR (MH "Advanced Practice Nurses/ED/EV/MA/MT/OG/ST/TD/UT") OR (MH "Cooperative Behavior/ED/EV/TD") OR (MH "Reflection/ED/EV/MT") OR (MH "Empowerment/ED/EV/TD") OR (MH "Social Participation/ED/EV/MA/ST/TD/UT") OR (MH "Problem-Based Learning") OR (TI “primary nurs*” OR AB “primary nurs*”) OR (TI “person centred care” OR AB “person centred care”) OR (TI “patient education” OR AB “patient education”) OR (TI "patient teaching" OR AB "patient teaching") OR (TI "counseling or guidance" OR AB "counseling or guidance") OR (TI "patient information" OR AB "patient information") OR (TI “family education” OR AB “family education”) OR (TI “holistic care” OR AB “holistic care”) OR (TI empath* OR AB empath*) OR (TI “patient collaboration” OR AB “patient collaboration”) OR (TI “patient involv*” OR AB “patient involv*”) OR (TI “patient participation” OR AB “patient participation”) OR (TI "nurse-patient relations" OR AB "nurse-patient relations") OR (MH "Total Patient Care Nursing/EV/ED/MA/MT/ST/TD/UT/SN") OR (MH "Patient Centered Care/EV/MA/ST/TD/UT") OR (MH "Continuity of Patient Care/EV/MA/ST") OR (MH "Patient Education/AM/ED/EV/MA/MT/OG/PF/ST/TD/UT") OR (MH "Professional-Family Relations/ED/EV/TD") OR (MH "Holistic Care/ED/EV/MA/MT/OG/NU/ST/TD/UT") OR (MH "Empathy/TD/EV/ED") OR (MH "Consumer Participation/ED/EV/MA/MT/OG/ST/TD/UT") OR (MH "Nurse-Patient Relations/ED/EV/PC/TD") OR (MH "Professional-Patient Relations/ED/EV/TD") OR (TI ebn OR AB ebn) OR (TI “evidence-based practice” OR AB “evidence-based practice”) OR (TI “evidence-based nursing” OR AB “evidence-based nursing”) OR (TI ebp OR AB ebp) OR (TI “nursing research” OR AB “nursing research”) OR (TI “workplace cultur*” OR AB “workplace cultur*”) OR (TI “work cultur*” OR AB “work cultur*”) OR (TI “team cultur*” OR AB “team cultur*”) OR (TI “organizational cultur*” OR AB “organizational cultur*”) OR (TI “nursing culture” OR AB “nursing culture”) OR (TI “cultural competency” OR AB “cultural competency”) OR (TI “collaborative decision*” OR AB “collaborative decision*”) OR (TI “shared decision making*” OR AB “shared decision making*”) OR (TI “organizational development” OR AB “organizational development”) OR (TI “practice change” OR AB “practice change”) OR (MH "Professional Practice, Evidence-Based/ED/EV/MA/MT/ST/TD/UT") OR (MH "Nursing Practice, Evidence-Based/ED/EV/MA/MT/ST") OR (MH "Research, Nursing/ED/EV/MT/OG/ST/TD") OR (MH "Clinical Nursing Research/TD/UT/ST/OG/MT/EV/ED") OR (MH "Education, Nursing, Research-Based/ED/EV/MA/OG/ST/TD/UT") OR (MH "Organizational Change/ED/EV/MA/MT/ST/UT") OR (MH "Organizational Culture+/ED/EV/MA/ST/TD/UT") OR (MH "Organizational Development/ED/EV/MA/MT/ST/TD/UT") OR (MH "Organizational Efficiency/ED/EV/MT/MA/ST/TD/UT") OR (MH "Organizational Structure/ED/EV/MT/ST/UT") OR (MH "Decision Making, Shared/ED/EV/MT") OR (MH "Organizational Development/ED/EV/MA/MT/ST/TD/UT") OR (TI "skill mix" OR AB "skill mix") OR (TI qualificat* OR AB qualificat*) OR (TI “organizational support*” OR AB “organizational support*”) OR (TI “power sharing” OR AB “power sharing”) OR (TI “shared leadership” OR AB “shared leadership”) OR (TI “shared governance” OR AB “shared governance”) OR (TI “transformational leadership” OR AB “transformational leadership”) OR (TI “workplace environment” OR AB “workplace environment”) OR (TI “risk management” OR AB “risk management”) OR (TI “physical environment” OR AB “physical environment”) OR (TI “social value*” OR AB “social value*”) OR (TI vision OR AB vision) OR (TI philosophy OR AB philosophy) OR (TI "shared value*" OR AB "shared value*") OR (TI strategy OR AB strategy) OR (MH "Skill Mix+") OR (MH "Leadership/ED/EV/TD") OR (MH "Shared Governance, Nursing") OR (MH "Nursing Management+/ED/EV/MA/MT/ST/TD/UT") OR (MH "Work Environment/ED/OG/ST/TD/UT") OR (MH "Risk Management/EV/MA/MT/ST/TD/UT") OR (MH "Social Values/TD/EV/ED") OR (MH "Values Clarification/ED/EV/TD") OR (MH "Vision/ED/EV/ST/MT") OR (MH "Philosophy, Nursing+") OR (MH "Value-Based Health Care/UT/TD/ST/MT/MA/EV/ED") OR (MH "Value-Based Purchasing/UT/TD/ST/MT/MA/HI/EV/ED") ) AND ( (TI “quality improvement*” OR AB “quality improvement*”) OR (TI “quality indicator*” OR AB “quality indicator*”) OR (TI “health care quality” OR AB “health care quality”) OR (TI wellbeing OR AB wellbeing) OR (TI “patient safety” OR AB “patient safety”) OR (TI “patient cultur*” OR AB “patient cultur*”) OR (TI “patient outcome*” OR AB “patient outcome*”) OR (TI “patient satisfaction” OR AB “patient satisfaction”) OR (TI “patient quality” OR AB “patient quality”) OR (TI “patient care” OR AB “patient care”) OR (TI “patient comfort” OR AB “patient comfort”) OR (TI “patient outcome assessment” OR AB “patient outcome assessment”) OR (MH "Quality of Health Care+/ED/EV/MA/MT/ST/TD/UT") OR (MH "Quality Improvement/EV/MA/MT/ST/TD/UT") OR (MH "Quality Assessment+/ED/MA/EV/MT/ST/TD/SN") OR (MH "Psychological Well-Being/CL/EV/ED/MT") OR (MH "Quality of Life/CL/ED/EV/MA/OG/PF/ST/TD/UT") OR (MH "Patient Safety/AN/CL/ED/EV/MT/RF/ST/TD/UT/PF") OR (MH "Patient-Reported Outcomes/AM/CL/ED/EV/MT/NU/OG/PF/ST/SN/TU/TD/UT") OR (MH "Outcomes (Health Care)/AM/ED/EV/MA/MT/ST/SN/TD/UT") OR (MH "Patient Satisfaction/CL/ED/EC/EV/MA/MT/PC/ST/SN") OR (MH "Quality Patient Care Scale/AE/CL/ED/EV/MT/OG/ST/TU/UT") OR (MH "Patient Care/ED/EV/MA/MT/NU/OG/ST/SN/TD/UT/MO/PF") OR (TI “personal satisfaction” OR AB “personal satisfaction”) OR (TI “job satisfaction” OR AB “job satisfaction”) OR (TI “work engagement” OR AB “work engagement”) OR (TI commitment OR AB commitment) OR (TI connected* OR AB connected*) OR (TI emancipat* OR AB emancipat*) OR (TI empower* OR AB empower*) OR (TI creativ* OR AB creativ*) OR (TI flourish* OR AB flourish*) OR (TI workflow* OR AB workflow*) OR (TI improvement OR AB improvement) OR (TI knowledge OR AB knowledge) OR (TI “professional autonomy” OR AB “professional autonomy”) OR (TI “personal growth” OR AB “personal growth) OR (MH "Personal Satisfaction/CL/ED/EV/PC/TD") OR (MH "Job Satisfaction/CL/ED/EV/MT/PC/TD") OR (MH "Work Engagement/CL/ED/EV/MA/MT/PH/PF/ST/SN/TD/UT") OR (MH "Commitment/TD/PC/EV/ED/CL") OR (MH "Empowerment/CL/ED/EV/TD/PC") OR (MH "Workflow/ED/CL/MA/MT/OG/ST/TD/UT") OR OR (MH "Nursing Knowledge/EV/ED/OG/ST/TD/UT") OR (MH "Outcomes of Education/ED/EV/OG/ST/TD/UT") OR (MH "Creativeness/ED/EV/MT/TD") OR OR (MH "Individuality/ED/EV/TD") OR (MH "Employee Performance Appraisal/ED/EV/MA/MT/ST/TD/UT") OR (MH "Employer-Employee Relations+/ED/EV/MT/ST/TD/UT") OR (MH "Professional Autonomy/ED/EV/MA/PC/ST/SN/TD/UT") OR (MH "Personal Growth/ED/EV/MT/OG") OR (TI benchmark OR AB benchmark) OR (TI “nursing outcome” OR AB “nursing outcome”) OR (TI “effective patient care” OR AB “effective patient care”) OR (TI “organizational commitment” OR AB “organizational commitment”) OR (MH "Benchmarking/ED/EV/MA/MT/ST/UT") OR (MH "Nursing Outcomes/AM/CL/ED/EV/MA/MT/ST/SN/TD/UT") ) ) AND AF german* NOT midwife* | | | |
| **GeroLit** | **Initial search conducted on: 11.06.2021** | | |
|  | **Search updated on: 01.03.2022** | | |
| Pflege AND (Pflegeentwick* OR Personalentwick* OR Kompetenzentwick* OR Kompenzerweiter* OR Weiterentwick* OR Transform* OR Veränder* OR Wandel* OR (Pflege AND Änder*) OR Innovation* OR (Praxis AND Theorie*) OR Implement* OR Methode* OR Evalu* OR PARIHS* OR Bildung* OR Wissen* OR Lern* OR Professionsentwick* OR Praxisentwick* OR Praxisveränder* OR (Praxis AND entwick*) OR (Praxis AND verbesser*) OR (Praxisvernetzung OR) Praxisnetz* OR Qualitätsverbesserung OR Qualitätsentwick* OR evidence-based Pflege OR evidenzbasierte Pflege OR Begleit* OR Unterstütz* OR Berat* OR Anleit* OR Change Agent OR Rollenentwick* OR ANP OR APN OR Advanced Practice Nurs* OR Advanced Nurs* Pract* OR Praxisanleit* OR Problemlösung* OR Förder* OR Zusammenarbeit* OR Reflexion* OR Empowerment OR Befähig* OR Ermächtig* OR Teilhabe* OR Primary Nurs* OR Bezugspflege* OR Primärpflege* OR Patientenorientier* OR patientenorientierte Pflege OR personenzentrierte Pflege OR person-zentrierte Pflege OR Person* orientierte Pflege OR Patientenzentriert* OR bedarfsorientierte Pflege* OR bedürfnisorientierte Pflege OR Patientenedukation OR Angehörigenedukation OR Interpersonale Beziehung OR Beziehung* Patient OR Beziehungsgestalt* OR Patientenbeziehung OR Empath* OR ganzheitliche Pflege OR Patientenzusammenarbeit OR holistisch* OR Eviden* OR Forschung* OR Kultur* OR shared decision making OR partizipative Entscheid* OR PEF OR (Skill AND Mix) OR (Grade AND Skill-Mix) OR Qualifikationsmix OR Mitarbeiterbeziehung* OR Mitarbeiterorientier* OR Organisationsunterstützung OR geteilte Führung OR Shared Leadership OR Shared Governance OR transformationale Führung OR (Innovation* AND Förder*) OR Risikomanagement OR Arbeitsplatzumgebung OR Arbeitsplatzgestalt* OR Werte OR Vision OR Strategie*) AND (Versorgungsqualität OR (Wohlbefinden AND Patient) OR Patientensicherheit OR (Patient AND Kultur) OR (Patient AND Outcome) OR (Kontinuität AND Versorgung) OR Versorgungskontinuität OR Patientenzufriedenheit OR Patientenversorgung OR Mitarbeiterzufriedenheit OR Mitarbeiterbindung OR (Pflege AND Verbunden*) OR Identifi* OR Engag* OR (Pflege AND Emanzip*) OR (Pflege AND Empowerment) OR (Pflege AND Befähig*) OR (Pflege AND Kreativ*) OR (Pflege AND Entfalt*) OR (Pflege AND Verbesser*) OR (Pflege AND Wissenserw*) OR Kosteneffizienz OR Benchmark OR Outcome OR (Effektivität* AND Patientenversorgung) OR organisationales Commitment OR organisationale Verbundenheit) | | | |
| **CareLit** | | **Initial search conducted on: 18.06.2021** | |
|  |  | **Search updated on: / (because the initial research showed that the other platforms also contained the hits)** | |
| Search with heading “Praxisentwicklung” | | | |
