# Supplementary material for: Characteristics of Implementing Practice Development in Germany: A National Scoping Review
Source: Health Sci Rep. 2025 Mar 18;8(3):e70546. doi: 10.1002/hsr2.70546 (PMC11915117; doi:10.1002/hsr2.70546)
Supplement: Supplementary file 2 — Supporting information. [file HSR2-8-e70546-s003.docx]

**SUPPLEMENT 2.** Overview of excluded reports through fulltext screening (Available in German)

| **Nr.** | **Autor*innen/Zeitschrift** | **Ausschlussgrund** |
| --- | --- | --- |
| 1 | N.N. (2014). "Qualitätsindikatoren in der Praxis, Teil 2 Der Entwicklungsprozess spielt eine wichtige Rolle." Care konkret(18): 9. | Volltext nicht verfügbar |
|  | *Kein Abstract vorhanden* | |
| 2 | Albert, U., et al. (2009). "Breast centers in Germany." Breast Care **4**(4): 225-230. | Nicht als Praxisentwicklungsprojekt deklariert |
|  | A decrease in medical practice variations in national breast cancer care has been shown to improve survival and the negative impact of the disease on affected women and their families. The following report describes the concert of efforts undertaken by the medical societies to optimize national breast cancer care by organizational centralization of multidisciplinary medical competence in certified breast centers (CBC), aiming to attain continual quality of health care by implementation of evidence- and consensus-based guidelines. Centralization and the systematic pursuit of organizational development by tracking guideline adherence using performance quality indicators over time demonstrate the feasibility and practicability of the implementation concept to bridge the gap between determined scientific best evidence and applied best practice. However, the proof of concept will remain pending until the data of the population-based cancer registries are analyzed for survival estimates. | |
| 3 | Bein, T. (2017). "Understanding intercultural competence in intensive care medicine." Intensive Care Medicine **43**(2): 229-231. | Nicht als Praxisentwicklungsprojekt deklariert |
|  | The article discusses the impact of globalization in critical care medicine. Topics include the effect of globalization which include the increasing interactions between participants in different cultural backgrounds which may also lead to cultural conflict, and decision making in clinical practice, the conflicting definition of death with respect to culture and religious perceptions, and presents the strategies to address the issue of conflict in the health care practice. | |
| 4 | Cotroneo, M. and M. Zimmer (2001). "Familienorientierte Pflege: Eine Strategie zur Verbesserung der Pflegequalität und zur Weiterentwicklung der Profession Pflege." Die Schwester, der Pfleger **40**(10): 809-813. | Nicht als Praxisentwicklungsprojekt deklariert |
|  | Versorgungsrelevante innovative Konzepte für die Weiterentwicklung der gesundheitlichen und pflegerischen Versorgung der Bevölkerung sollen hier am Beispiel des Steuerungskonzeptes "Family Health Nurse" vorgestellt werden. Zur Umsetzung der in GESUNDHEIT21, dem Rahmenprogramm der Weltgesundheitsorganisation (WHO), geforderten Qualifizierung von Fachkräften für gesundheitliche Aufgaben wurde von der WHO im Bereich des Pflegewesens das Family-Health-Nurse-Konzept (FHN) entwickelt. Dieses Konzept bietet die Möglichkeit, u. a. neue Handlungsfelder in der ambulanten Pflege zu entwickeln, da die gesundheitspolitischen Maxime "ambulant vor stationär" und "Rehabilitation vor Pflege" nichts an Aktualität verloren haben. Eine authentische und alltägliche Geschichte. | |
| 5 | Czakert, J., et al. "[Patient safety in home care: A review of international recommendations]." Z Evid Fortbild Qual Gesundhwes **135**: 18-26. | Nicht als Praxisentwicklungsprojekt deklariert |
|  | BACKGROUND: In recent years there has been a growing trend towards nursing care at home in general as well as towards intensive home care being provided by specialized home care services in Germany. However, resulting challenges for patient safety have rarely been considered. Against this background we aimed to explore whether international recommendations for patient safety in home care in general and in intensive home care in particular already exist and how they can stimulate further practice development in Germany. METHODS: A review of online English documents containing recommendations for patient safety in intensive home care was conducted. Available documents were analyzed and compared in terms of their form and content. RESULTS: Overall, a small number of relevant documents could be identified. None of these documents exclusively refer to the intensive home care sector. Despite their differences, however, the analysis of four selected documents showed similarities, e. g., regarding specific topics of patient safety (communication, involvement of patients and their relatives, risk assessment, medication management, qualification). Furthermore, strengths and weaknesses of the documents became apparent: e. g., an explicit understanding of patient safety, a literature-based introduction to safety topics or an adaptation of the recommendations to the specific features of home care were occasionally lacking. CONCLUSIONS: This document analysis provides interesting input to the formal and content-related development of specific recommendations and to practice development in Germany to improve patient safety in home care. | |
| 6 | Dieffenbach, S., et al. (2001). Einrichtungsübergreifende Vernetzung zur Sicherung einer kontinuierlichen Patientenversorgung, Bern, Huber. | Volltext nicht verfügbar |
|  | *Kein Abstract vorhanden* | |
| 7 | Döhner, H. (1999). "Pflege vernetzt: Bausteine zur verbesserten Zusammenarbeit auf struktureller und individueller Ebene." Evangelische Impulse **21**(2): 21-25. | Nicht als Praxisentwicklungsprojekt deklariert |
|  | *Kein Abstract vorhanden* | |
| 8 | Dummert, A. (2012). "Begleiter mit Führungskompetenz: Die Bedeutung von Leadership in der Praxisanleitung." Padua : die Fachzeitschrift für Pflegepädagogik **7**(3): 116-121. | Nicht als Praxisentwicklungsprojekt deklariert |
|  | Wissen und fachliche Fähigkeiten weiter zu entwickeln, reicht für eine nachhaltige Verbesserung der Patientenversorgung nicht aus. Leadership ist gefragt, damit Praxisanleitung effektiv wirkt. Das Leadership-Challenge Konzept von Kouzes und Posner kann hierbei als Vorbild dienen. | |
| 9 | Eberhardt, D. (2021). "Praxisentwicklung braucht auch eine pädagogische Basis " PADUA **7**(3): 107. | Nicht als Praxisentwicklungsprojekt deklariert |
|  | *Kein Abstract vorhanden* | |
| 10 | Elsbernd, A. and F. Hohloch (2010). Damit die Pflege zu Hause gelingen kann: ein generationenverbindendes Wohnprojekt: Modellprojekt zur Entwicklung eines Pflegenetzwerks. Lage, Jacobs. | Nicht als Praxisentwicklungsprojekt deklariert |
|  | In diesem Buch wird über ein Modellprojekt zur Erprobung neuer, wohnortnaher Versorgungsformen für pflegebedürftige Menschen berichtet. Das Forschungsprojekt war angesiedelt in einem (neuen) Wohnquartier, in dem bewusst der Schwerpunkt auf ein gelingendes generationenverbindendes Zusammenleben gelegt wurde. Im Verlauf des Projektes wurde ein sogenanntes „Pflegenetzwerk“ entwickelt, das das Ineinanderwirken von (pflege-)professionellen Hilfen, pflegerischer Infrastruktur, Pflege in Familien, Pflege in der Nachbarschaft und Engagement Freiwilliger ermöglichen und fördern sollte. Im Buch werden die Komplexität des Aufbaus von wohnortnahen Versorgungsstrukturen für pflegebedürftige Menschen praxisnah beschrieben und die tiefgreifenden Veränderungen im Selbstverständnis der Beteiligten diskutiert. Darüber hinaus haben die Autorinnen aus der Sicht von (befragten) pflegebedürftigen Menschen des Wohnquartiers Indikatoren zur Beschreibung des Hilfe- und Pflegeprozesses im außerfamiliären Netzwerk und zur Beschreibung des Sozialen Kapitals erarbeitet. Dieser Ansatz verdeutlicht, dass ambulante Versorgungsmodelle die Heterogenität der Lebenslagen von pflegebedürftigen Bürgerinnen und Bürgern aufgreifen und deshalb flexible, individualisierte und im Gemeinwesen verankerte Hilfe- und Unterstützungsleistungen aufbauen müssen. Die Leserinnen und Leser werden auf eine Entdeckungsreise mitgenommen, die erleben lässt, dass wohnortnahe Pflege nur dann gelingen kann, wenn durchdachte, gesellschaftliche Trends berücksichtigende, mit Bürger/innen gemeinsam erarbeitete und fachlich abgestimmte Modelle entwickelt werden. Dieses Buch ist für interessierte Bürgerinnen und Bürger ebenso von Nutzen wie für Planer und Anbieter von pflegerisch-gesundheitlicher Versorgung. | |
| 11 | Flerchinger, C. (2001). Zusammenführung von ärztlichen und pflegerischen Berufsgruppen in Stationsteams zur Verbesserung von Motivation und Effizienz, Bern, Huber. | Nicht als Praxisentwicklungsprojekt deklariert |
|  | *Kein Abstract vorhanden* | |
| 12 | Frings, R. (2016). Schreiben mit Demenzkranken: Impuls zur Belebung der beeinträchtigten Kommunikation: Erinnerungsalbum mit Nutzwert für Angehörige und andere pflegende Personen. Berlin, Springer**:** 127-136. | Nicht als Praxisentwicklungsprojekt deklariert |
|  | In diesem Aufsatz geht es um die Erfahrungen aus der Schreibwerkstatt für Menschen mit Demenz. Sie fertigen zusammen mit ihren Angehörigen in begleiteter Zusammenarbeit Tagebücher an. Mit den Tagebüchern möchte das Projekt dem bis ins späte Stadium der Krankheit vorhandenen Wunsch nach Kommunikation einen Ausdruck verleihen. | |
| 13 | Gomes, V., et al. (2016). "Developing an innovative, integrated care pathway for PMV patients." International Journal of Integrated Care (IJIC) **16**(6): 1-2. | Nicht als Praxisentwicklungsprojekt deklariert |
|  | Background: In the traditional care pathway for prolonged mechanically ventilated (PMV) patients, the patients often progress from an intensive care unit (ICU) directly to their home or to an unspecialised nursing home setting. In these settings, specialised offerings for PMV patients such as round-the-clock respiratory rehabilitation and weaning programmes are usually not being offered. Therefore in the traditional pathway, PMV patients do not receive the integrated rehabilitation and therapy programmes they require. Moreover, patients returning home without professional support are a challenge for the family caregivers, often resulting in unplanned patient readmission to the hospital and ICU. In addition to this suboptimal care situation, PMV patients cause very high resource consumption for hospitals: whereas PMV patients account for ten percent of ICU patients, they can consume up to fifty percent of the ICU resources. Care coordination between stakeholders: In contrast to hospital ICUs, dedicated service offerings outside of the hospital setting have the potential to improve the care that PMV patients receive. In 2011, Linde AG - Healthcare developed an innovative programme for PMV patients offering an integrated, standardized care path outside a hospital's ICU including a patient's own home. The programme spans a variety of operating models to meet individual needs at different stages of the care continuum. The patients can easily move between the operational models of this path, depending on the complexity of care and the support required. The mission of this programme is to manage the whole PMV patient pathway by ensuring highest quality of care in an efficient and effective manner in order to establish an optimal care service offering for PMV patients. To achieve this mission it is essential to integrate the patient pathway between different care providers and decision makers. In addition to the optimisation of care to PMV patients, the optimisation of treatment costefficiency in the fragmented setting of public and private providers is the key argument for Linde's innovative care pathway. For being able to attract stakeholders along the path, reimbursement needs to be tailored such that reimbursement is not solely reduced but rather optimized to combine sustainable financial results with optimal patient results i.e. in the form of sharing cost savings, reduced hospitalization and exacerbations as well as by means of telehealth/- monitoring. In order to bring together, manage and improve all the aspects of integration between the stakeholders in the innovative care programme, the Integrated Care Pathway Project (ICPP) assessment tool was developed. It is based on six pillars: Leadership, Interdisciplinary Teams, Coordination of Care, Quality and Safety Assurance, Financial Management and Reimbursement and Patient-centred Care. When looking at integration aspects in the care programme, there are two types of integration to consider: the internal integration between the different operating models of the care pathway of the single provider (Linde Healthcare) as well as the external integration/collaboration with external partners (hospitals, other service providers, community). Assessing internal and external integration levels: Although there are many examples of integration assessment tools for the healthcare market, they are mainly specific for public sector integration assessment or specific disease management pathway integration assessment and therefore do not exactly match the Linde programme's needs. Based on a thorough literature review and the above mentioned main pillars and principles of integration, a specific assessment tool for internal and external integration was developed. This integration assessment tool comprises over 200 questions (internal and external integration each) and is divided into the six main pillars. Data is collected using an interview methodology and subsequent analysis is based on the worldwide standard for RADAR from the European Foundation for Quality Management (EFQM) wit it enabler and results areas [1]. For the classification of assessment results, four levels of integration are foreseen: Minimal integration, basic collaboration, and close collaboration - partly integrated and fully integrated respectively [2]. Early results of the first assessments are available with regard to the level of integration. The external integration assessment suggests that the majority of pillars are within the scope of 'basic collaboration'. Concerning the internal integration, the integration level is higher, reaching 'close collaboration - partly integrated'. Based on further evaluations, integration improvement projects will be started. | |
| 14 | Gültekin, J. E. und A. Liebchen (2003). Pflegevisite und Pflegeprozess: Theorie und Praxis für die stationäre und ambulante Pflege. Stuttgart, Kohlhammer. | Nicht als Praxisentwicklungsprojekt deklariert |
|  | *Kein Abstract vorhanden* | |
| 15 | Hahn, S. (2012). "Herausforderungen und Chancen: Evidenzbasierte Pflege in Praxis und Forschung." Psychiatrische Pflege heute : Fachzeitschrift für die psychiatrische Pflege **18**(2): 65-68. | Nicht in Deutschland durchgeführt |
|  | Die sogenannte evidenzbasierte Pflege und Gesundheitsversorgung gibt immer wieder Anlass zu Diskussionen und stellt Praxis und Forschung vor große Probleme. Medizinische, therapeutische und pflegerische Leistungen sollen gleichzeitig wirksam, zweckmäßig und wirtschaftlich erbracht werden. Eine Herausforderung - ja - doch die Autorinnen zeigen, welche Chancen eine evidenzbasierte Praxis für eine fundierte und nachhaltige Praxisentwicklung birgt. | |
| 16 | Hahn, S. (2019). "Gute Pflege ist kein Zufall: Pflegequalität verbessern in Zusammenarbeit von Praxis und Forschung." NOVAcura **50**(9): 21-25. | Nicht in Deutschland durchgeführt |
|  | *Kein Abstract vorhanden* |  |
| 17 | Haslbeck, J. W. (2011). "Selbstmanagement und empowerment fördern: chronisch Kranke." Pro Alter **43**(6): 42-46. | Nicht als Praxisentwicklungsprojekt deklariert |
|  | *Kein Abstract vorhanden* | |
| 18 | Hauss, A., et al. (2015). "Erfolgreich im Kampf gegen Dekubitus und Sturz: klinisches Qualitäts- und Risikomanagement." Die Schwester, der Pfleger **54**(7): 42-44. | Nicht als Praxisentwicklungsprojekt deklariert |
|  | Das Risikomanagement zum Schutz vor Dekubitus und Sturz, das die Charité - Universitätsmedizin Berlin implementiert hat, ist kürzlich mit dem ersten Preis für Patientensicherheit 2015 des Aktionsbündnisses Patientensicherheit ausgezeichnet worden. Im folgenden Beitrag erläutern die Preisträger ihren klugen Ansatz, der nachweislich zu geringeren Dekubitus- und Sturzraten geführt hat. (Vorlage) S1 - Gemeinsamer Bibliotheksverbund (GBV) / Verbundzentrale des GBV (VZG) S2 - Deutsches Zentrum für Altersfragen, Berlin | |
| 19 | Hoffmann, A., et al. (2021). "PERSONENZENTRIERT UND ZUKUNFTSORIENTIERT." Die Schwester, der Pfleger(5): 32-36. | Nicht als Praxisentwicklungsprojekt deklariert |
|  | Praxisentwicklung in der Pflege Die Praxisentwicklung ist ein kontinuierlicher Prozess, der im Zusammenspiel verschiedener Methoden die Pflege effektiver gestaltet und eine personenzentrierte Versorgung zum Ziel hat. Dazu bedarf es Veränderungen der Praxis und der Individuen in einer Einrichtung. Leitend sind gemeinsame Werte und Visionen sowie eine eigene Zieldefinition der personenzentrierten Pflege. | |
| 20 | Hoffmann, S. (2017). "Die Analyse unerwünschter Ereignisse als Beitrag zur Sicherheitskultur im Kontext der Praxisentwicklung." Pflege: die wissenschaftliche Zeitschrift für Pflegeberufe **30**(6): 339-346. | Nicht in Deutschland durchgeführt |
|  | Hintergrund: Die Analyse unerwünschter Ereignisse ist eine wirksame Patientensicherheitsmassnahme. Ziel: Wir zeigen, wie Pflegeexpertinnen zur Analyse unerwünschter Ereignisse mit dem _Learning from Defects-Tool_ (LFD-Tool) befähigt wurden. Methode: Für die Implementierung des Tools wählten wir eine umfassende Strategie, die sowohl am Sicherheitswissen der Pflegexpertinnen als auch an ihrer Einstellung zum Thema _Patientensicherheit_ ansetzte. Dabei wurde die Kultur der Praxisentwicklung berücksichtigt. Die Implementierungsstrategie und das LFD-Tool werden beschrieben. Ergebnisse: Pflegeexpertinnen berichten durch die Anwendung des LFD-Tools einen Kompetenzaufbau zum Thema _Patientensicherheit_. Die Anwendung des Tools unterstützte die gemeinsame Reflexion von unerwünschten Ereignissen in den Pflegeteams. Schlussfolgerungen: Die Analyse unerwünschter Ereignisse fördert das gemeinsame, arbeitsplatznahe Lernen. Pflegende können durch die Nutzung des LFD-Tools einen wichtigen Beitrag zur aktiven Gestaltung der Sicherheitskultur in einem Spital leisten. | |
| 21 | Kapp, H. (2005). "Nach aktuellem Wissensstand handeln: Eine gute Zusammenarbeit zwischen Pflegedienst, Ärzten und Angehörigen verbessert ein Therapieerfolg." Häusliche Pflege **14**(8): 30-32. | Volltext nicht verfügbar |
|  | *Kein Abstract vorhanden* | |
| 22 | Kirchen-Peters, S. "[Gerontopsychiatric Consultation-Liaison Service--pioneers wait for imitators]." Z Gerontol Geriatr **41**(6): 467-474. | Nicht als Praxisentwicklungsprojekt deklariert |
|  | *Kein Abstract vorhanden* | |
| 23 | Kirchen-Peters, S. (2003). "Die Situation Demenzkranker verbessern!: Ein Beispiel dafür, wie praxisnahe Forschung und konzeptgeleitete Umsetzung ineinandergreifen können." ISO-Mitteilungen(2): 41-49. | Nicht als Praxisentwicklungsprojekt deklariert |
|  | The treatment and care of individuals with dementia, who are admitted to general hospitals due to somatic diseases, often leads to crisis situations which not only compromise the therapeutic benefit, but also can be followed by avoidable institutionalization in nursing homes or psychiatric hospitals. With the model project "Gerontopsychiatric Consultation-Liaison Service" in Kaufbeuren a comprehensive solution to this problem has been developed. A multiprofessional team consisting of a medical specialist, nurses specialized in psychiatry and an occupational therapist supports the health personnel by giving assistance and advice. It also provides contact persons for patients and their families. The effectiveness analysis proved that the work of this service has positive effects on both patient care and costs. Moreover a structural and process analysis provided data about the structure of patients and services as well as about the problems which arose during the setting up of the new service. Potential "imitators" can use this information in the implementation of similar services. | |
| 24 | Kohlen, H. (2011). "Care transformations: attentiveness, professional ethics and thoughts towards differentiation." Nurs Ethics **18**(2): 258-261. | Nicht als Praxisentwicklungsprojekt deklariert |
|  | *Kein Abstract vorhanden* | |
| 25 | Körner, M., et al. (2018). "A patient-centred team-coaching concept for medical rehabilitation." Journal of Interprofessional Care **32**(1): 123-126. | Nicht als Praxisentwicklungsprojekt deklariert |
|  | Team coaching enhances teamwork and subsequently improves patient-centredness in medical rehabilitation clinics. Even though interprofessional teamwork is regarded as a crucial factor in medical rehabilitation, to date no evaluated team-coaching approaches are available for improving interprofessional teamwork in medical rehabilitation in Germany. Based on a systematic literature search and interviews with staff, managers, and patients of rehabilitation clinics, we developed a team-coaching approach that is standardized in its process but based on the individual needs and requests of each clinic. It takes a systemic perspective and is goal-oriented and solution-focused. The approach mainly serves to provide impulses to make use of resources within the team and to support a self-directed organisational learning process. It is manualized and can, therefore, be used by professionals aiming to improve interprofessional teamwork in their clinic. A multi-centre, cluster-randomized controlled study that was conducted to evaluate the team-coaching approach showed positive results. Team organization, knowledge integration, and responsibility can be improved, and, therefore, the implementation of the patient-centred team-coaching approach in interprofessional rehabilitation teams can be recommended. | |
| 26 | Krcmar, C. R. (1997). "Bedeutung der Pflegeforschung in der Praxis." Pflege Zeitschrift **50**(8). | Nicht als Praxisentwicklungsprojekt deklariert |
|  | *Kein Abstract vorhanden* | |
| 27 | Kricheldorff, C. and E. Bubolz-Lutz (2013). Das Modell Pflegebegleitung: Vernetzung, Kompetenzentwicklung und Empowerment als Prinzipien bei der Begleitung pflegender Angehöriger. Zürich, Seismo**:** 244-262. | Nicht als Praxisentwicklungsprojekt deklariert |
|  | *Kein Abstract vorhanden* | |
| 28 | Kühme, B. and E. Narbei (2019). "Aus der Praxis und für die Praxis: Entwicklung von pflegedidaktisch reflektierten Transferaufgaben." PADUA **14**(1). | Nicht als Praxisentwicklungsprojekt deklariert |
|  | *Kein Abstract vorhanden* | |
| 29 | Kummer, K., et al. "[Communication about incontinence between affected individuals and health care providers - the patients' perspective]." Z Gerontol Geriatr **41**(4): 267-273. | Nicht als Praxisentwicklungsprojekt deklariert |
|  | Communication is a key competence for medical and nursing health care providers alike. However, there appear to be areas of "speechlessness" regarding specific medical problems, including the "taboo" disease incontinence. There is a lack of scientific data regarding incontinence in the context of communication among patients and health care professionals.The descriptive study was designed to provide insights how female and male patients alike perceive communications about incontinence with doctors and nurses, respectively. 22 structured interviews were conducted and taped with 16 elderly female and 6 male patients (age >/= 60 years, mean: 81 years, demented patients excluded, n = 19 analysable), and five interviews each with doctors and nurses within the setting of a rehabilitation hospital for geriatric patients (not reported here). We used published methodology to interpret the diversity of patients' verbal communications to the female interviewer.Results suggest that patients wish to find a relationship based on sympathy, empathy and trust. If incontinence is addressed, it is by doctors, not patients. Statements by patients suggest that they differentiate between expectations addressing the professional level of medical care, and a level perhaps best described as the quality of the personal relationship between the patient and doctor. However, these twofold expectations were not distinguishable regarding nursing professionals, they were perceived on a "personal care level" only, not the professional level regarding incontinence. Independent of professional affiliation, patients expect empathy, understanding, and respect. They selectively criticize manners of (some) nurses and lack of understandable transfer of information by doctors. The gender of the care providers was not an issue for patients regarding communication about incontinence. The preliminary results show that there is room for improvement for better communication regarding incontinence. However, areas affected appear to differ between nursing and medical professionals. | |
| 30 | Linschoten, M., et al. (2016). "The Pink Passkey®" - ein Zertifikat für die Verbesserung der Akzeptanz von LSBT*I-Plegebedürftigen in Pflegeeinrichtungen. Wiesbaden, Springer VS**:** 227-241. | Nicht als Praxisentwicklungsprojekt deklariert |
|  | *Kein Abstract vorhanden* | |
| 31 | Marzi, I. (2013). "Quality improvement in trauma care." European Journal of Trauma & Emergency Surgery **39**(1): 1-2. | Nicht als Praxisentwicklungsprojekt deklariert |
|  | *Kein Abstract vorhanden* | |
| 32 | Matthaeus-Kraemer, C. T., et al. (2015). "Barriers and supportive conditions to improve quality of care for critically ill patients: A team approach to quality improvement." Journal of Critical Care **30**(4): 685-691. | Nicht als Praxisentwicklungsprojekt deklariert |
|  | Purpose: Despite the fact that Quality Improvement (QI) teams are widespread tools for improving performance in medical settings, little is known about what makes teams effective and successful. The goal of this study was to identify barriers and supportive conditions for QI teams to implement an effective and successful QI project to improve quality of care.  Materials and methods: Multicenter expert interviews with 17 team leaders were conducted in a cluster randomized trial. Interviews were based on a semistructured interview guide and were recorded and transcribed. Qualitative analysis was performed according to the principles of grounded theory.  Results: The major findings of our study can be summarized in a framework of conditions that support the implementation of changes by QI teams. This framework can be divided into 5 core categories: the availability of external support, an interdisciplinary QI team, staff characteristics such as dedicated employees who are aware and experienced, and generally supportive structural circumstances. Furthermore, the interviewees reported that changes should be disseminated through, for example, repeating key elements or addressing employees directly.  Conclusions: Using a grounded theory-based qualitative approach, we identified a framework of conditions supportive of QI-related change, which can help project initiators to create environments that are supportive of change. | |
| 33 | Mayer-Amberg, N., et al. (2015). "An Integrated Care Initiative to Improve Patient Outcome in Schizophrenia." Front Psychiatry **6**: 184. | Nicht als Praxisentwicklungsprojekt deklariert |
|  | The optimal treatment of schizophrenia patients requires integration of medical and psychosocial inputs. In Germany, various health-care service providers and institutions are involved in the treatment process. Early and continuous treatment is important but often not possible because of the fragmented medical care system in Germany. The Integrated Care Initiative Schizophrenia has implemented a networked care concept in the German federal state of Lower Saxony that integrates various stakeholders of the health care system. In this initiative, office-based psychiatrists, specialized nursing staff, psychologists, social workers, hospitals, psychiatric institutional outpatient's departments, and other community-based mental health services work together in an interdisciplinary approach. Much emphasis is placed on psychoeducation. Additional efforts cover socio-therapy, visiting care, and family support. During the period from October 2010 (start of the initiative) to December 2012, first experiences and results of quality indicators were collected of 713 registered patients and summarized in a quality monitoring report. In addition, standardized patient interviews were conducted, and duration of hospital days was recorded in 2013. By the end of 2012, patients had been enrolled for an average of 18.7 months. The overall patient satisfaction measured in a patient survey in June 2013 was high and the duration of hospital days measured in a pre-post analysis in July 2013 was reduced by 44%. Two years earlier than planned, the insurance fund will continue the successfully implemented Integrated Care Initiative and adopt it in the regular care setting. This initiative can serve as a learning case for how to set up and measure integrated care systems that may improve outcomes for patients suffering from schizophrenia. | |
| 34 | Mensdorf, B. "[Step by step to nursing competence--2: Body care: an underestimated responsibility]." Pflege Z **60**(6): 342-344. | Nicht als Praxisentwicklungsprojekt deklariert |
|  | *Kein Abstract vorhanden* | |
| 35 | Mette, M., et al. (2019). "Implementing MIA - Mannheim's interprofessional training ward: first evaluation results." GMS J Med Educ **36**(4): Doc35. | Nicht als Praxisentwicklungsprojekt deklariert |
|  | Project description: In Germany there is great interest in better preparing learners in the health care professions for interprofessional (IP) collaboration on IP training wards. On the MIA, Mannheim's interprofessional training ward, medical students, nursing apprentices and physiotherapy (PT) trainees learn and practise real patient care in a team under supervision. The concept of the MIA, its implementation and the first evaluation results are reported. During the 2017/18 academic year, 201 medical students, 72 nursing apprentices and 33 PT trainees completed their mandatory placements on the MIA, which they evaluated online at the end of the placement (questions on the organisation of the MIA placement, learning gains, supervision, participant satisfaction, personal insights). The data was analysed according to frequency for each health care profession separately using the Kruskal-Wallis test for comparing the evaluation data between the three participant groups. Results: The response rate was 45% (104 medical students, 16 nursing apprentices, 19 PT trainees). 64% of the medical students considered the placement too short. For 70% of the nursing apprentices, the number of patients to be treated was too high. The supervision by the facilitators was adequate. There were often IP contacts. Professional and IP learning gains were rated high. IP learning took place mainly in personal conversations and on IP ward rounds. IP communication/collaboration was mentioned most often as an important insight gained from the placement. Discussion: The implementation of the MIA concept is considered successful. The learning objectives were achieved. The structured daily routine on the ward with its IP elements promotes IP collaboration and helps to minimise difficulties in the clinical placement, which - often for the first time - demands that the participants manage patient care in an accountable manner. Conclusion: Placements on IP training wards in the education of health care professionals can be a good preparation for practising optimal patient care in the future. | |
| 36 | Mihaljevic, A. L., et al. (2018). "Heidelberger Interprofessionelle Ausbildungsstation (HIPSTA): a practice- and theory-guided approach to development and implementation of Germany's first interprofessional training ward." GMS J Med Educ **35**(3): Doc33. | Nicht als Praxisentwicklungsprojekt deklariert |
|  | Background: Deficits in care and impaired patient-safety have been linked to inefficient interprofessional collaborative practice. Interprofessional training wards (IPTW) are an interprofessional educational intervention which aim to enable students and trainees from different health professions to work self-responsibly in order to manage the medical treatment and rehabilitation of real-life patients together as an interprofessional team. We aimed to develop and implement Germany´s first IPTW at the department of Surgery at Heidelberg University Hospital. Methods: The Kern cycle was used to develop an ITPW curriculum. Practical as well as theoretical considerations guided the design of the IPTW. Common project management tools including blueprinting and RASCI (Responsibility, Approval, Support, Consultation, Information) matrix were applied. Results: Since April 2017, 7 cohorts of students and trainees have had four-week long placements on HIPSTA. They run the IPTW in early and late shifts. Nursing and medical facilitators are supporting the IP team as needed. Learning objectives are operationalized as EPAs (entrustable professional activities) and interprofessional learning goals. Since initiation only minor modifications to the curriculum have been necessary and satisfaction of students/trainees, facilitators and patients is high. Conclusion: IPTWs can be established and run in the German health care system even in a complex clinical setting. The early involvement of all professions in a steering group seems to be key to success. Nursing and medical facilitators are of utmost importance for daily routine. The experiences outlined here could help others aiming to implement IPTWs at their sites. IPTWs might address a number of hitherto unaddressed educational needs. Trial registration: Not applicable. | |
| 37 | Moers, M. (2007). "Pflegetheorien heute: Wie können sie die Praxisentwicklung fördern? : Teil 2." Die Schwester - Der Pfleger **46**(1): 70-73. | Nicht als Praxisentwicklungsprojekt deklariert |
|  | *Kein Abstract vorhanden* | |
| 38 | Moers, M. and D. Schaeffer (2006). "Theorieentwicklung. Pflegetheorien heute: Wie können Sie die Praxisentwicklung fördern? Teil 1." Die Schwester, der Pfleger(12). | Nicht als Praxisentwicklungsprojekt deklariert |
|  | *Kein Abstract vorhanden* | |
| 39 | Morgenstern, U. and K. Ketelhut (2017). Brain-Gym bei Demenz: Ein aktivierendes Pflegekonzept zur Konzentrationssteigerung und Verbesserung der Alltagskompetenzen in ambulanten Betreuungseinrichtungen. Hamburg, Verlag Dr. Kovač**:** 113-133. | Nicht als Praxisentwicklungsprojekt deklariert |
|  | *Kein Abstract vorhanden* | |
| 40 | Neumann, E.-M. (1996). Emanzipation der Pflege durch Verwissenschaftlichung der Pflegepraxis. Bremen, Altera Verl.ges.**:** 43-54. | Nicht als Praxisentwicklungsprojekt deklariert |
|  | *Kein Abstract vorhanden* | |
| 41 | Offermann, C. (2015). "Best Practice Pflege: Reflexionsprozesse für eine nachhaltige Pflegepraxis in kompetenz- und praxisorientierten Weiterbildungen." Pädagogik der Gesundheitsberufe **2**(4): 25-30. | Nicht in Deutschland durchgeführt |
|  | *Kein Abstract vorhanden* | |
| 42 | Peters, S., et al. (2019). "Views of healthcare professionals on training for and delivery of a fatigue self-management program for persons with multiple sclerosis." Disability & Rehabilitation **41**(23): 2792-2798. | Nicht in Deutschland durchgeführt |
|  | Purpose: To explore the experiences and perspectives of the healthcare professionals who were trained to and delivered "Minimise Fatigue, Maximise Life" (MFML), a patient-centered group-based fatigue self-management program for persons with multiple sclerosis. Methods: A qualitative descriptive study with semi-structured individual interviews at two time points. Data were analyzed for themes. Six healthcare professional facilitators who were trained to and delivered "Minimise Fatigue, Maximise Life" participated in a first interview, and five in a second. Participants were all female, aged between 23 and 66 years old and either occupational therapists or physiotherapists. Results: Two themes were evident in the data. The first, "Reciprocity," showed how the healthcare professionals were trained to deliver MFML, then reciprocated in the program delivery as active participants, which then provided feelings of personal reward and expansion of their usual practice. The second, "Enhancements," encompassed suggested directions for future training and deliveries of the program. Conclusion: This study suggests that multidimensional patient centered interventions also benefit the healthcare professionals who provide them because it expands their practice. Healthcare professionals who recognize the benefits of innovative and patient-centered interventions, supports both the patients with whom they work, and adds value to the health services they provide. Healthcare professionals who undergo training to facilitate delivery of self-management programs, which are based in an empowerment model, report an enhancement or expansion of their traditional practice. An empowerment-based program delivered in a group situation encourages and facilitates people to draw on their own and peers' knowledge and expertise to problem solve for self-management. Healthcare professional education should facilitate the healthcare professional's learning, and ability and willingness to acknowledge the richness in knowledge and expertise held by their patients. | |
| 43 | Quaschning, K., et al. (2013). "Analyzing the effects of shared decision-making, empathy and team interaction on patient satisfaction and treatment acceptance in medical rehabilitation using a structural equation modeling approach." Patient Education & Counseling **91**(2): 167-175. | Keine Beteiligung der Profession Pflege |
|  | OBJECTIVES: The aims of the study are: (1) To develop and test a theory-based model for the predictive power of 'Shared decision making (SDM)', 'Empathy' and 'Team interaction' for 'Patient satisfaction' and 'Treatment acceptance'. (2) To identify mediating effects of 'Compliance' and 'Satisfaction with decision'. METHODS: Within a multi-center cross-sectional study (11 inpatient rehabilitation clinics at different indication fields), the model was evaluated in descriptive and structure analytical terms based on survey data of N=402 inpatients. RESULTS: The structural equation model proved to exhibit an appropriate data fit. A high proportion of variance of 'Patient satisfaction' (61%) and 'Treatment acceptance' (67%) can be predicted by 'SDM', 'Empathy', 'Satisfaction with decision' and 'Team interaction'. While no mediating effects were found for the two subcomponents of 'Compliance' ('Patient cooperation', 'Adherence'), 'Satisfaction with decision' showed a full mediation for 'Treatment acceptance' and a partial mediation for 'Patient satisfaction'. CONCLUSION: 'Team interaction' should be considered as an important predictor of process and patient-centered outcome characteristics. PRACTICE IMPLICATIONS: The current findings can be used to derive measures as well as interventions to optimize the organization of participatory care within teams in order to strengthen patient centeredness and to ensure a high quality of care. | |
| 44 | Ruhe, M. C., et al. (2011). "Appreciative Inquiry for Quality Improvement in Primary Care Practices." Quality Management in Health Care **20**(1): 37-48. | Nicht in Deutschland durchgeführt |
|  | *Kein Abstract vorhanden* | |
| 45 | Satzinger, W., et al. (2004). "Der Patienten-Begleitbogen: Der Versuch, die Kommunikation bei der Pflegeüberleitung zu verbessern." Forum Sozialstation **28**(128): 34-37. | Nicht als Praxisentwicklungsprojekt deklariert |
|  | *Kein Abstract vorhanden* | |
| 46 | Schilder, M. (2010). "Zur Bedeutung der klinischen Pflegewissenschaft für eine forschungsbasierte Praxisentwicklung." Pflege & Gesellschaft **15**(1). | Nicht als Praxisentwicklungsprojekt deklariert |
|  | *Kein Abstract vorhanden* | |
| 47 | Schubert, B. and M. Wrobel (2009). "Identifizierung von Hindernissen, die die Implementierung von Forschungswissen in die Pflegepraxis hemmen." Pflegewissenschaft **11**(6): 343-351. | Nicht als Praxisentwicklungsprojekt deklariert |
|  | *Kein Abstract vorhanden* | |
| 48 | Sieger, M. and W. Kunstmann (2003). "Versorgungskontinuität durch Pflegeüberleitung?: Ergebnisse eines Modellprojekts." Pflegemagazin **4**(6): 8-15. | Nicht als Praxisentwicklungsprojekt deklariert |
|  | *Kein Abstract vorhanden* | |
| 49 | Sowinski, C. and H. Esser (2004). Planungshilfe Bezugs(personen)pflege: personenzentrierte Pflege auch in "traditionellen" Pflegeeinrichtungen ; Arbeitshilfe für einen organisatorischen Wandel von der Funktions- zur Bezugspflege ; [im Rahmen des BMGS-Modellprogramms zur Verbesserung der Versorgung Pflegebedürftiger]. Köln, Kuratorium Deutsche Altershilfe. | Nicht als Praxisentwicklungsprojekt deklariert |
|  | *Kein Abstract vorhanden* | |
| 50 | von Eiff, W. (2006). "Ideenmanagement im Krankenhaus Status quo und Entwicklungsmöglichkeiten in der Praxis." Das Krankenhaus **98**(6). | Nicht als Praxisentwicklungsprojekt deklariert |
|  | *Kein Abstract vorhanden* | |
| 51 | Willems, L. M., et al. (2019). "Tools for your stroke team: adapting crew-resource management for acute stroke care." Practical Neurology (BMJ Publishing Group) **19**(1): 36-42. | Nicht als Praxisentwicklungsprojekt deklariert |
|  | Crew-resource management is an approach to work and training that focuses on non-technical skills and strategies to prevent human error in complex procedures. It was initially termed 'cockpit-resource management' and developed for aviation in the 1970s after several severe accidents; it has contributed to a measurable increase in flight safety. In recent years, this approach has been successfully implemented in other high- reliability environments; surgical disciplines have made particular use of crew-resource management strategies and training, with resulting reduced mortality rates. The stepwise implementation of different crew-resource management strategies in stroke care at our tertiary stroke centre has helped to speed up process times significantly, and to improve patient safety and staff satisfaction. Here, we summarise our experience in adapting different crew-resource management tools to acute stroke care, sharing specific tools that have proven valuable in our hands, and we encourage colleagues to implement such strategies in acute stroke care. | |
| 52 | Wirth, A. et al. (2020). "Interprofessionelles Lernen zum Thema Patienten-Selbstbestimmung: Resultate aus der Praxisperspektive der Careum Summer School 2019." Krankenpflege SBK = Soins infirmières = Cure infermieristiche **113**(11): 32-34. | Nicht in Deutschland durchgeführt |
|  | Die Careum Summer School (CSS) leistet mit den beiden Kernthemen Interprofessionalität und Selbstbestimmung einen Beitrag, um die Zusammenarbeit in der Praxis zu reflektieren und die Versorgungsqualität zu verbessern. Der vorliegende Artikel lotet die Potenziale des Lernformats CSS zur Umsetzung der Projektideen in der interprofessionellen Praxis aus, mit dem Ziel, die Praxisentwicklung vor Ort voranzutreiben. | |
| 53 | Wohkittel, C. and D. Spürk (2011). "Der Erwerb beruflicher Handlungskompetenz. Die Entwicklung eines Curriculums für den Lernstandort Praxis." Pflegewissenschaft **13**(4). | Nicht als Praxisentwicklungsprojekt deklariert |
|  | *Kein Abstract vorhanden* | |
| 54 | Wöhrmann, E. (2013). "Mit starken Teams in neue Strukturen: Verbesserungspotenziale." Altenheim **52**(7): 20-23. | Nicht als Praxisentwicklungsprojekt deklariert |
|  | *Kein Abstract vorhanden* | |
| 55 | Zimmermann, D. (2004). "Versorgungskontinuität sichern: Implementierung eines pflegerischen Entlassungsmanagements, Teil 1." Die Schwester, der Pfleger **43**(10): 736-741. | Nicht als Praxisentwicklungsprojekt deklariert |
|  | *Kein Abstract vorhanden* | |
